# Quantitative Proteomics Reveals that Hsp90 Inhibition Dynamically Regulates Global Protein Synthesis in Leishmania mexicana

**DOI:** 10.1128/mSystems.00089-21

**Published:** 2021-05-11

**Authors:** Karunakaran Kalesh, Sandeep Sundriyal, Hirunika Perera, Steven L. Cobb, Paul W. Denny

**Affiliations:** a Department of Chemistry, Durham University, Durham, United Kingdom; b Department of Pharmacy, Birla Institute of Technology and Science, Pilani, India; c Department of Biosciences, Durham University, Durham, United Kingdom; Oxford Nanopore Technologies

**Keywords:** Hsp90, tanespimycin, *Leishmania mexicana*, quantitative proteomic mass spectrometry, BONCAT, *Leishmania*

## Abstract

Heat shock protein 90 (Hsp90) is a conserved molecular chaperone responsible for the folding and maturation of nascent proteins. Hsp90 is regarded as a master regulator of protein homeostasis in the cell, and its inhibition affects the functions of a large array of client proteins. The classical Hsp90 inhibitor tanespimycin has shown potent antileishmanial activity. Despite the increasing importance of Hsp90 inhibition in the development of antileishmanial agents, the global effects of these inhibitors on the parasite proteome remain unknown. By combining tanespimycin treatment with bioorthogonal noncanonical amino acid tagging (BONCAT) metabolic labeling and isobaric tags for relative and absolute quantitation (iTRAQ)-based quantitative proteomic mass spectrometry, for the first time, we robustly profiled the relative changes in the synthesis of hundreds of parasite proteins as functions of dose and duration of the inhibitor treatment. We showed that Hsp90 inhibition dynamically regulates nascent protein synthesis in Leishmania mexicana, with many chaperones and virulence factors showing inhibitor concentration- and treatment duration-dependent changes in relative expression. Many ribosomal proteins showed a downregulation upon severe Hsp90 inhibition, providing the first protein-level evidence that Hsp90 inhibition affects the protein synthesis capacity of the ribosome in this organism. We also provide an unbiased target validation of tanespimycin in L. mexicana using live parasite photoaffinity labeling with a novel chemical probe and quantitative proteomic mass spectrometry. We showed that the classical Hsp90 inhibitor not only engages with its presumed target, Hsp83-1, in L. mexicana promastigotes but also affects multiple proteins involved in protein synthesis and quality control in the parasite. This study defines the *Leishmania* parasites’ response to Hsp90 inhibition at the level of nascent global protein synthesis and provides a rich resource for future studies on *Leishmania* spp. biology and antileishmanial drug development.

**IMPORTANCE** *Leishmania* spp. are the causative agents of leishmaniasis, a poverty-related disease, which is endemic in >90 countries worldwide, affecting approximately 12 million people, with an estimated 700,000 to 1 million new cases and around 70,000 deaths annually. Inhibitors of the chaperone protein Hsp90 have shown promising antileishmanial activity. However, further development of the Hsp90 inhibitors as antileishmanials is hampered by a lack of direct information of their downstream effects on the parasite proteome. Using a combination of mass spectrometry-based quantitative proteomics and chemical and metabolic labeling, we provide the first protein-level evidence that Hsp90 inhibition affects global protein synthesis in *Leishmania*. We also provide the precise relative quantitative changes in the expressions of hundreds of affected proteins as functions of both the concentration and duration of the inhibitor treatment. We find that *Leishmania* regulates its ribosomal proteins under Hsp90 inhibition while a set of virulence factors and chaperones are preferentially synthesized.

## Abstract (precis)

*Leishmania* spp. are the causative agents of leishmaniasis, a poverty-related disease, which is endemic in >90 countries worldwide, affecting approximately 12 million people, with an estimated 700,000 to 1 million new cases and around 70,000 deaths annually. Inhibitors of the chaperone protein Hsp90 have shown promising antileishmanial activity.

## INTRODUCTION

Conserved from bacteria to mammals, heat shock protein 90 (Hsp90) acts as a master regulator of protein homeostasis in the cell by facilitating the maturation and activation of a large array of client proteins involved in cell signaling, proliferation, and survival (1). Hsp90 is a therapeutic target of significant interest for the treatment of cancers (2) as well as several parasitic diseases, including leishmaniasis (3), Chagas disease (4), and malaria (5). The protozoan parasite *Leishmania* sp., the causative agent of leishmaniasis, has been found to be critically reliant on Hsp90 for its stress adaptation and survival during its complex digenetic life cycle (3, 6, 7). However, the biological functions of Hsp90 in *Leishmania*, and the proteins it regulates in the parasite, remain largely unknown.

Unlike in higher eukaryotes, the genomes of unicellular *Leishmania* spp. contain no confirmed genes for transcriptional regulation or *cis*-acting regulatory DNA sequences such as gene promoters and enhancers. The genes in *Leishmania* are organized into long polycistronic transcription units that encode functionally unrelated proteins (8). From the polycistronic transcripts, individual mature mRNAs are generated by 5′ *trans*-splicing of a small capped spliced leader sequence and 3′ polyadenylation (9). In the absence of promoter-mediated gene regulation, *Leishmania* spp. rely on posttranscriptional mechanisms such as mRNA stability, protein translation, protein folding, degradation, and posttranslational modifications (PTMs) for regulating gene expression (10).

As an essential protein, Hsp90 null mutants are not viable; therefore, reverse genetic methods are not suitable for unravelling the functions of this important protein (7, 11). Therefore, a chemical inhibition approach would be ideal for systematically analyzing the consequences of disrupting Hsp90 at the proteome level in the *Leishmania* sp. parasite. The macrocyclic benzoquinone antibiotic tanespimycin, also known as 17-allylamino-17-demethoxygeldanamycin (17-AAG), is the most studied inhibitor of Hsp90 (12). Tanespimycin was previously shown to potently inhibit the growth of *Leishmania* spp. parasites both *in vitro* and *in vivo* (3). As Hsp90 regulates many proteins, we sought for a direct protein-level characterization of the downstream effects of Hsp90 inhibition on protein synthesis in the *Leishmania* parasite.

Deep sequencing of ribosome-protected mRNA fragments, termed ribosome profiling, is emerging as a powerful method for analyzing protein translation (13). A recent study employed ribosome profiling to monitor the effects of Hsp90 inhibition on protein synthesis in Leishmania donovani parasites (14). In ribosome profiling, the association of mRNAs with translating ribosome is used as an indirect means to estimate protein translation. A fundamental assumption in ribosome profiling is the uniformity of translation elongation rates among all mRNAs in the cell (13). Although this is generally true, up to 20-fold variation in translation elongation rates among the open reading frames (ORFs) has been reported (15). The differences in the translation elongation rates between mRNAs is attributed to various factors, including codon biases, availability of tRNAs, translational cofolding of polypeptides, and presence of positively charged amino acids in the nascent polypeptide sequence (15–19). Translation elongation of mRNAs encoding ribosomal proteins (RPs) was reported to occur at a lower rate that for other mRNAs with similar ribosome densities (15). Ribosome profiling also suffers from many technical limitations. These include contaminating footprint-sized fragments of rRNAs causing erroneous readouts of protein translation (20) and artifacts caused by translation elongation inhibitors, as they alter the local distributions of ribosomes on an mRNA (21). Additionally, ribosome profiling requires a large input sample (22), as at any given time point, only a fraction of mRNAs are associated with the ribosomes. In principle, every experimental step in ribosome profiling has the potential to generate erroneous data output (23). Proteins compared to mRNAs are more robust for sample handling, and quantitative proteomic mass spectrometry (MS)-based methods provide a more direct and more reliable alternative for measuring protein translation (24–29). A direct measurement of the nascent proteins is particularly important under stress conditions, as recent studies indicate a poor correlation between indirect measurements of protein synthesis from ribosome profiling and direct measurements using quantitative proteomic MS (29). In the present study, using quantitative proteomic MS, we show that Hsp90 inhibition causes a global repression in protein synthesis in Leishmania mexicana. Importantly, ribosome profiling is prone to bias when assessing translation under conditions of global repression (30, 31).

We coupled tanespimycin treatment with bioorthogonal noncanonical amino acid tagging (BONCAT) metabolic labeling (25) and isobaric tags for relative and absolute quantitation (iTRAQ) (32) quantitative proteomic MS. Our results robustly identified, for the first time, the dose- and time-dependent effects of tanespimycin treatment on the synthesis of several key parasite proteins beyond its main target, Hsp90 (known as Hsp83-1 in L. mexicana), pointing to a polypharmacology-based mechanism of action for the compound. We find that L. mexicana responds to Hsp90 inhibition by selectively downregulating its ribosomal protein synthesis while a preferential synthesis of several virulence factors and chaperones occurs. The study defines the downstream effectors of Hsp90 inhibition in L. mexicana and provides precise relative quantitation of the effect of dose- and time-depended Hsp90 inhibition on hundreds of nascent parasite proteins. Additionally, we evaluated for the first time the target engagement of tanespimycin in L. mexicana using competitive affinity-based protein profiling (AfBPP) (33–35) with a novel minimalist terminal alkyne photoaffinity probe that closely mimics tanespimycin and revealed the protein target spectrum of the Hsp90 inhibitor in the parasite.

## RESULTS

### Quantitative proteomic MS profiling of tanespimycin-induced changes in global nascent protein synthesis in L. mexicana.

**FIG 1:**
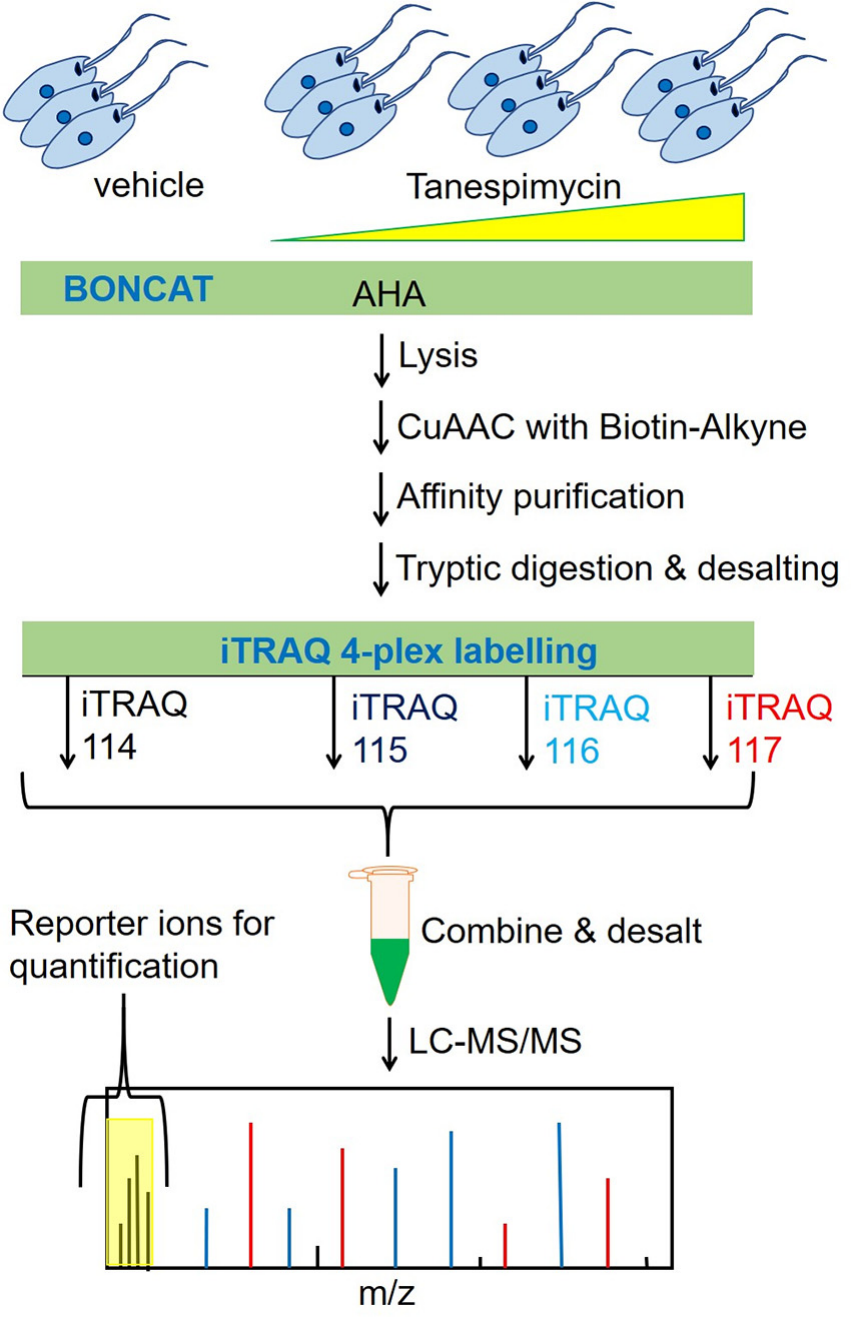
Schematic overview of the Hsp90 inhibition combined with BONCAT metabolic labeling and iTRAQ-based quantitative proteomic MS for systematic profiling of effect of Hsp90 inhibition on protein synthesis in *Leishmania*.

**FIG 2:**
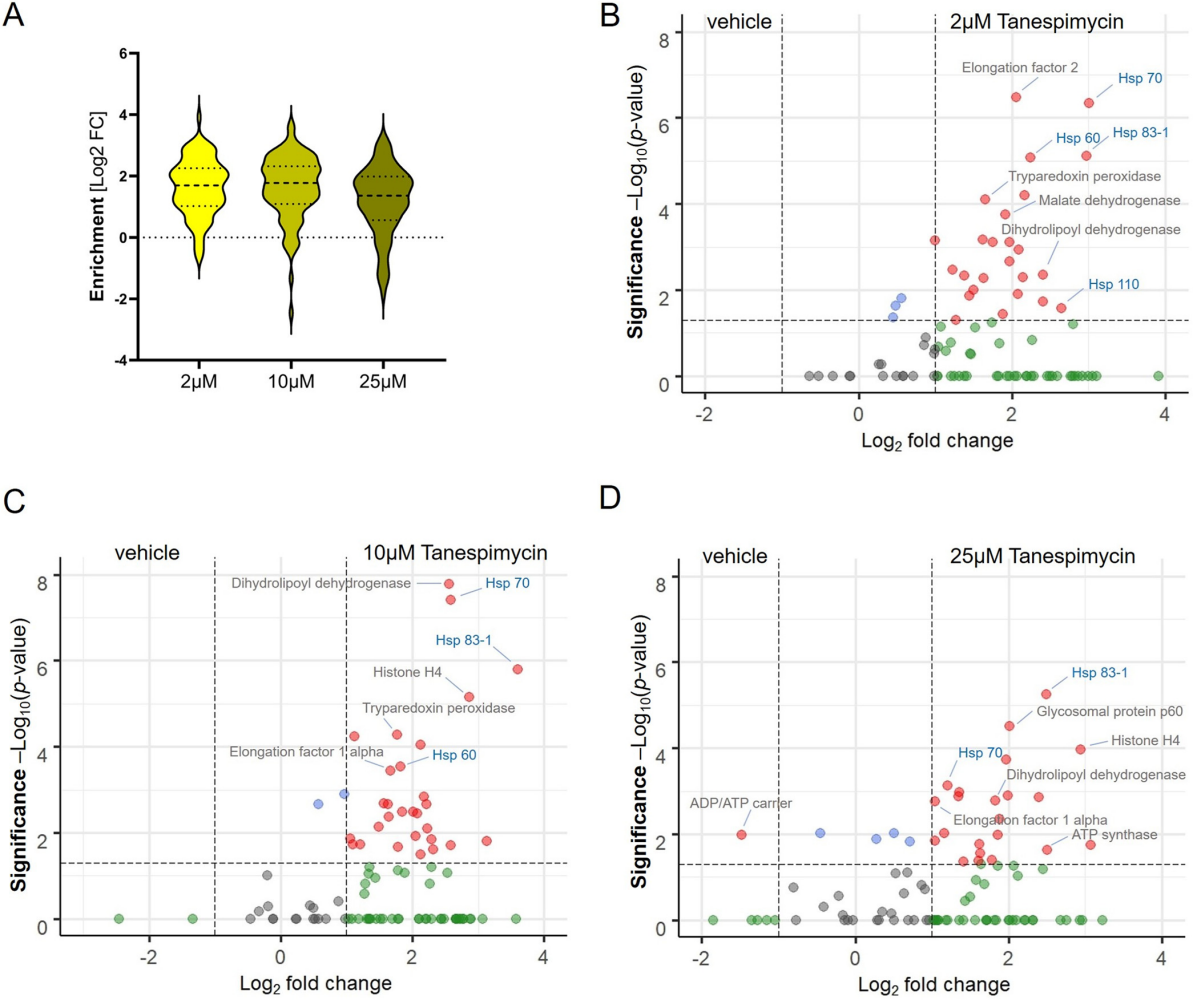
Characterization of effect of Hsp90 inhibition on nascent protein synthesis in L. mexicana promastigotes. (A) Violin plot demonstrating the tanespimycin concentration-dependent dynamic changes in global nascent protein synthesis. Volcano plots show significant enrichment of newly synthesized proteins at 2 μM (B), 10 μM (C), and 25 μM (D) tanespimycin treatment over that with vehicle (DMSO) treatment across three replicates. A modified *t* test with permutation-based FDR statistics was applied (250 permutations, FDR = 0.05) to compare tanespimycin-treated and vehicle-treated groups.

We employed a combination of BONCAT and iTRAQ labeling-based quantitative proteomic MS (36) for a direct measurement of effect of tanespimycin treatment on protein synthesis in L. mexicana promastigotes (Fig. 1). We treated the parasites with three different concentrations (2 μM, 10 μM, and 25 μM) of tanespimycin and vehicle (dimethyl sulfoxide [DMSO]) in three replicates for 2 h in a methionine-free Schneider’s medium supplemented with azidohomoalanine (AHA). The short treatment window and the tanespimycin concentration range were chosen to robustly profile differentially expressed proteins in the early periods of Hsp90 inhibition without causing an impact on parasite viability. Following the treatments, the parasites were lysed, and the whole-cell lysates were subjected to a click reaction (37) with a biotin-alkyne capture reagent and affinity enriched on NeutrAvidin-agarose resin. After on-bead tryptic digestion, the samples were labeled with iTRAQ 4-plex reagents and analyzed by liquid chromatography tandem mass spectrometry (LC-MS/MS) (see Table S1 in the supplemental material). Only subtle changes were observed in the nascent proteins from 2 μM to 10 μM concentration of tanespimycin. However, a higher concentration (25 μM) of the inhibitor caused a statistically significant global decrease in nascent protein synthesis, revealing that Hsp90 inhibition significantly affects protein translation in this organism even during a short 2-h treatment window (Fig. 2A). More importantly, the quantitative proteomic MS results, as presented in the volcano plots (Fig. 2B, C and D), showed tanespimycin concentration-dependent altered expression of several newly synthesized proteins (NSPs).

10.1128/mSystems.00089-21.6 TABLE S1 Newly synthesized L. mexicana proteins at 2, 10, and 25 μM tanespimycin treatment for 2 h. Download Table S1, XLSX file, 0.1 MB. Copyright © 2021 Kalesh et al. 2021 Kalesh et al. https://creativecommons.org/licenses/by/4.0/ This content is distributed under the terms of the Creative Commons Attribution 4.0 International license.

Chaperone Hsp70, a major component of the Hsp90 foldosome complex, is consistently identified among the top abundantly expressed NSPs upon tanespimycin treatment. Hsp90 inhibition in human cancer cells was previously reported to cause undesirable activation of the master heat shock transcription factor 1 (HSF1), which in turn causes increased expression of the cytoprotective chaperones, mainly Hsp70, leading to counterproductive cytoprotective heat shock responses during cancer therapy (38, 39). Our results indicate that similarly to that in human cancer cells, quantifiable increased expression of Hsp70 is a hallmark of Hsp90 inhibition in L. mexicana. More importantly, our study revealed, for the first time, that expression of not only Hsp70 but also a panel of other parasite proteins increased upon inhibition of Hsp90. The statistically significant differentially expressed proteins among these NSPs (represented by red filled circles on the top right of each volcano plot in Fig. 2B, C, and D) are potential molecular mediators of the parasite’s adaptation to the Hsp90 inhibition stress. Our results also showed that Hsp90 (Hsp83-1) is among the most increasingly expressed NSPs upon its own inhibition in L. mexicana. The iTRAQ 4-plex labeling approach additionally enabled quantitative comparison of the relative changes in the expressions of the NSPs as a function of the concentration of the Hsp90 inhibitor (Table S1). At the lower 2 μM tanespimycin treatment, the increased Hsp70 expression was more prominent, while with increased tanespimycin, the Hsp70 expression relative to that of Hsp83-1 was found to decrease (Fig. S1). The Hsp90 inhibitor concentration-dependent relative changes in abundances were also visible in several other NSPs (Fig. S1).

10.1128/mSystems.00089-21.1 FIG S1 Effect of tanespimycin concentration on the synthesis of selected proteins in L. mexicana promastigotes revealed by iTRAQ labelling-based quantitative proteomic mass spectrometry. The log2 fold change in abundance of the proteins upon tanespimycin treatment (2, 10, and 25 μM concentrations for 2 h) with respect to that with vehicle (DMSO) treatment are shown. Download FIG S1, PDF file, 0.3 MB. Copyright © 2021 Kalesh et al. 2021 Kalesh et al. https://creativecommons.org/licenses/by/4.0/ This content is distributed under the terms of the Creative Commons Attribution 4.0 International license.

### Temporal effect of Hsp90 inhibition on global nascent protein synthesis in L. mexicana.

**FIG 3:**
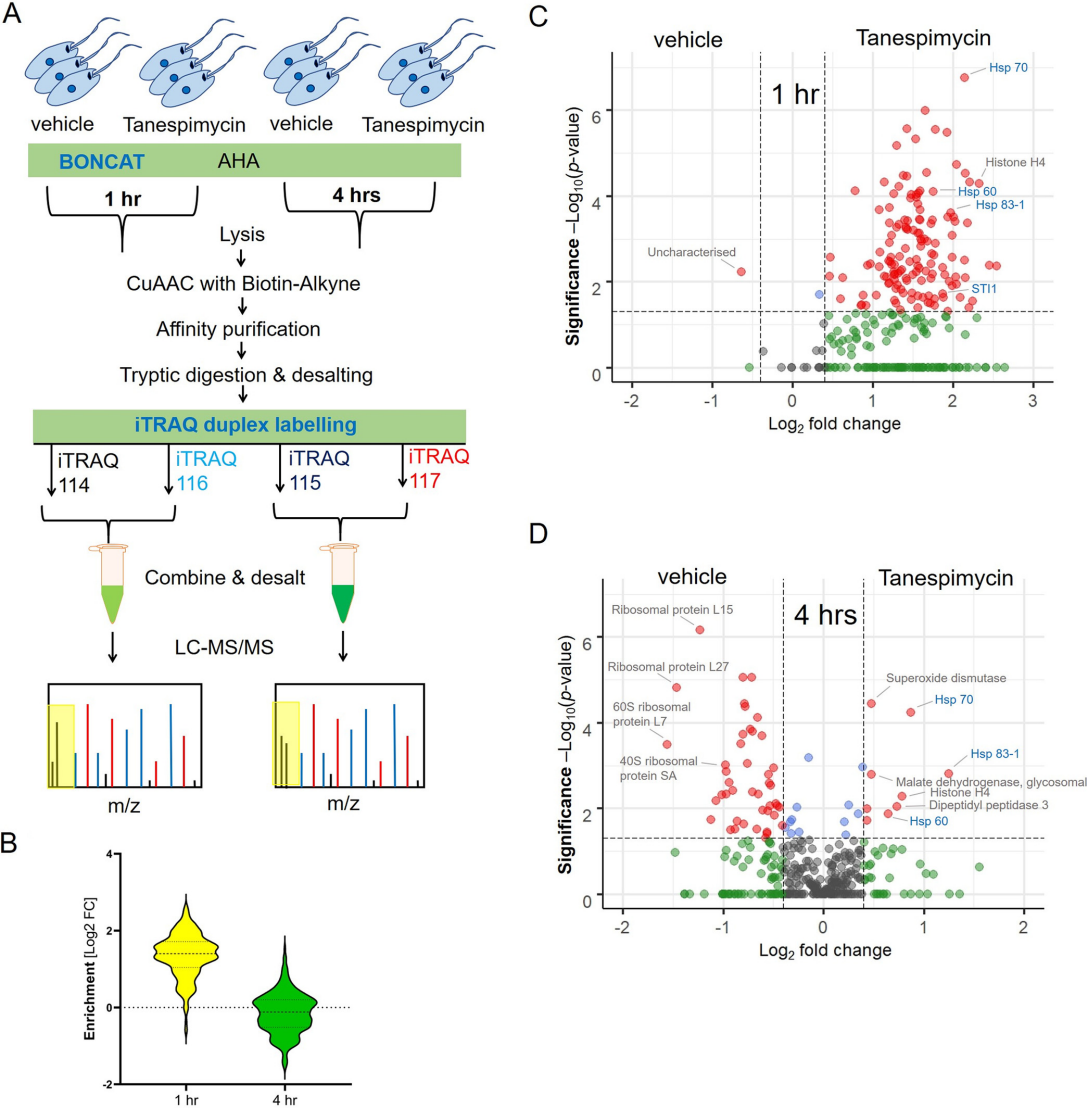
Temporal effect of Hsp90 inhibition on nascent protein synthesis in L. mexicana promastigotes. (A) Schematic of the workflow involving tanespimycin treatment combined with BONCAT metabolic labeling and iTRAQ-quantitative proteomic MS. (B) Violin plot demonstrating the significant decrease of global protein synthesis at 4 h compared to that at 1 h of treatment. Volcano plots show enrichment of newly synthesized proteins at 1 h (C) and 4 h (D) of tanespimycin treatment (50 μM) over that with vehicle (DMSO) treatment across three replicates. A modified *t* test with permutation-based FDR statistics was applied (250 permutations, FDR = 0.05) to compare tanespimycin-treated and vehicle-treated groups.

To assess the temporal effect of tanespimycin treatment on global protein synthesis in L. mexicana, the compound treatment was combined with the BONCAT-iTRAQ quantitative proteomic MS workflow using a fixed concentration of 50 μM but varying the treatment duration (1 h and 4 h) (Fig. 3A). The maximum treatment duration was limited to 4 h, as it was reported earlier as a suitable time window for avoiding irreversible effects of tanespimycin on *Leishmania* (40). Despite its high potency, the parasite-killing effect of tanespimycin take place slowly, and even 100 μM tanespimycin did not cause a significant effect on L. mexicana viability within the 4-h treatment period (data not shown). Two separate iTRAQ duplex experiments were performed in triplicates to take into account the temporal effects of protein synthesis in the tanespimycin-untreated samples. A total of 158 and 200 NSPs were identified after filtering away contaminants, endogenous biotinylated proteins, and proteins identified with less than 2 unique peptides for the 1-h and 4-h treatment windows, respectively (see Tables S2 and S3). Of these Hsp90 inhibition-responsive NSPs, statistically significant enrichment across the three replicates was identified for 156 and 73 proteins for the 1-h and 4-h treatments, respectively. As shown in the violin plots (Fig. 3B), a dramatic decrease in the global synthesis of new proteins was observed at the 4-h Hsp90 inhibition window compared to that for the 1-h treatment, revealing the temporal effect of severe Hsp90 inhibition on protein translation in this organism. An increase in the synthesis of many proteins with respect to the vehicle treatment was observed at the initial 1-h Hsp90 inhibitor treatment period (Fig. 3C). However, as the inhibition proceeded to 4 h, repression in the synthesis of many RPs was observed (Fig. 3D), indicating that the Hsp90 inhibition affects the state of the ribosome in L. mexicana as reported in the case of mammalian cells (41, 42).

10.1128/mSystems.00089-21.7 TABLE S2 Newly synthesized L. mexicana proteins at 1 h tanespimycin (50 μM) treatment. Download Table S2, XLSX file, 0.1 MB. Copyright © 2021 Kalesh et al. 2021 Kalesh et al. https://creativecommons.org/licenses/by/4.0/ This content is distributed under the terms of the Creative Commons Attribution 4.0 International license.

10.1128/mSystems.00089-21.8 TABLE S3 Newly synthesized L. mexicana proteins at 4 h tanespimycin (50 μM) treatment. Download Table S3, XLSX file, 0.1 MB. Copyright © 2021 Kalesh et al. 2021 Kalesh et al. https://creativecommons.org/licenses/by/4.0/ This content is distributed under the terms of the Creative Commons Attribution 4.0 International license.

Protein-protein interaction network analysis of the Hsp90 inhibition-affected nascent proteins using STRING database (43) of a related Leishmania major species showed known interactions within the network (see Fig. S2). Gene ontology (GO) analysis of the statistically significant downregulated proteins revealed structural constituent of ribosome (*P* value, 2.93e^−45^) and translation (*P* value, 7.29e^−40^) as extremely enriched molecular function and biological process GO terms, respectively (see Fig. S3A). Importantly, the quantitative proteomic MS approach enabled not only the identification but also the quantitation of relative changes in the expression levels of the affected RPs. Despite the repression in RP synthesis, increased expression of a smaller set of L. mexicana proteins occurred under Hsp90 inhibition. The statistically significant proteins (red filled circles at upper right portion of Fig. 3D) preferentially synthesized by the parasite under severe Hsp90 inhibition include known virulence factors superoxide dismutase (SOD) (44), Hsp70 (45), and dipeptidyl peptidase 3 (46), important metabolic enzymes, and histone H4. Gene ontology (GO) analysis of the statistically significant upregulated proteins revealed unfolded protein binding (*P* value, 3.77e^−6^) and protein folding (*P* value, 2.53e^−7^) as significantly enriched molecular function and biological process GO terms, respectively (Fig. S3B). Collectively, the nascent proteins preferentially synthesized by L. mexicana under Hsp90 inhibition revealed in this study are functionally important proteins that the parasite relies upon to mitigate the cytotoxic effects of Hsp90 inhibition.

10.1128/mSystems.00089-21.2 FIG S2 Protein-protein interaction network of the Hsp90 inhibitor-affected L. mexicana nascent proteome. Network analysis was performed using the publicly available STRING database of L. major strain Friedlin and visualized using Cytoscape. Upregulated and downregulated proteins are presented as green and yellow nodes, respectively. The STRING database score of each interaction has been embedded in the width of the network edges. Download FIG S2, PDF file, 0.02 MB. Copyright © 2021 Kalesh et al. 2021 Kalesh et al. https://creativecommons.org/licenses/by/4.0/ This content is distributed under the terms of the Creative Commons Attribution 4.0 International license.

10.1128/mSystems.00089-21.3 FIG S3 Gene ontology term enrichment of L. mexicana nascent proteins that showed decreased expression (A) and increased expression (B) upon tanespimycin treatment relative to the predicted total proteome of the organism. The GO terms were refined and visualized using REVIGO software. Download FIG S3, PDF file, 0.6 MB. Copyright © 2021 Kalesh et al. 2021 Kalesh et al. https://creativecommons.org/licenses/by/4.0/ This content is distributed under the terms of the Creative Commons Attribution 4.0 International license.

### *In situ* photoaffinity labeling combined with quantitative proteomic MS revealed targets of tanespimycin in L. mexicana.

**FIG 4:**
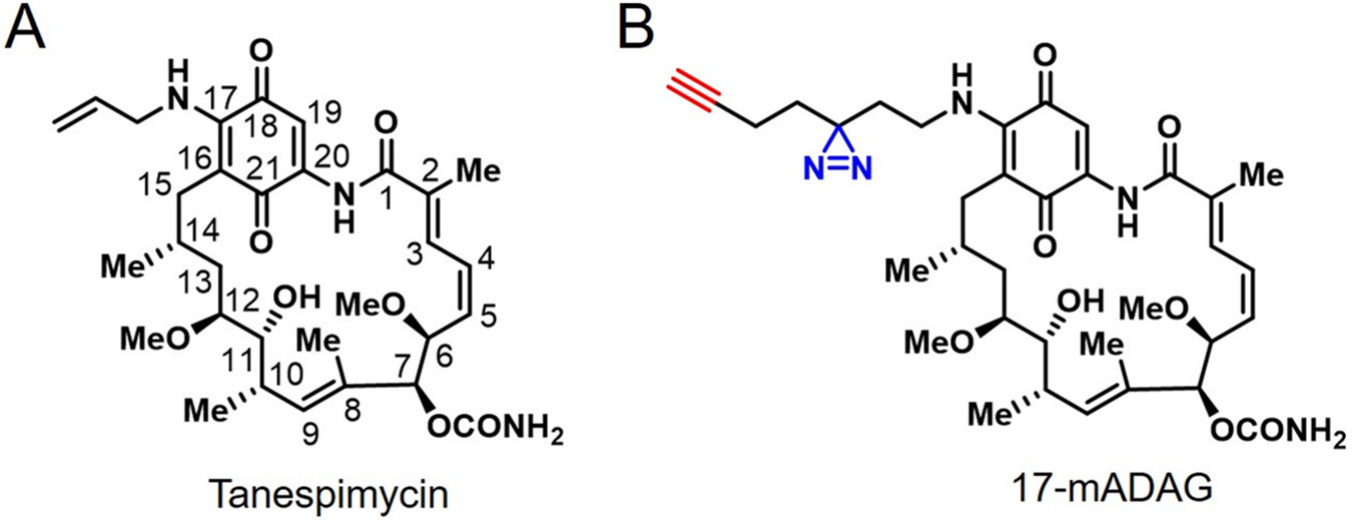
Chemical structures of tanespimycin (A) and 17-mADAG (B).

**FIG 5:**
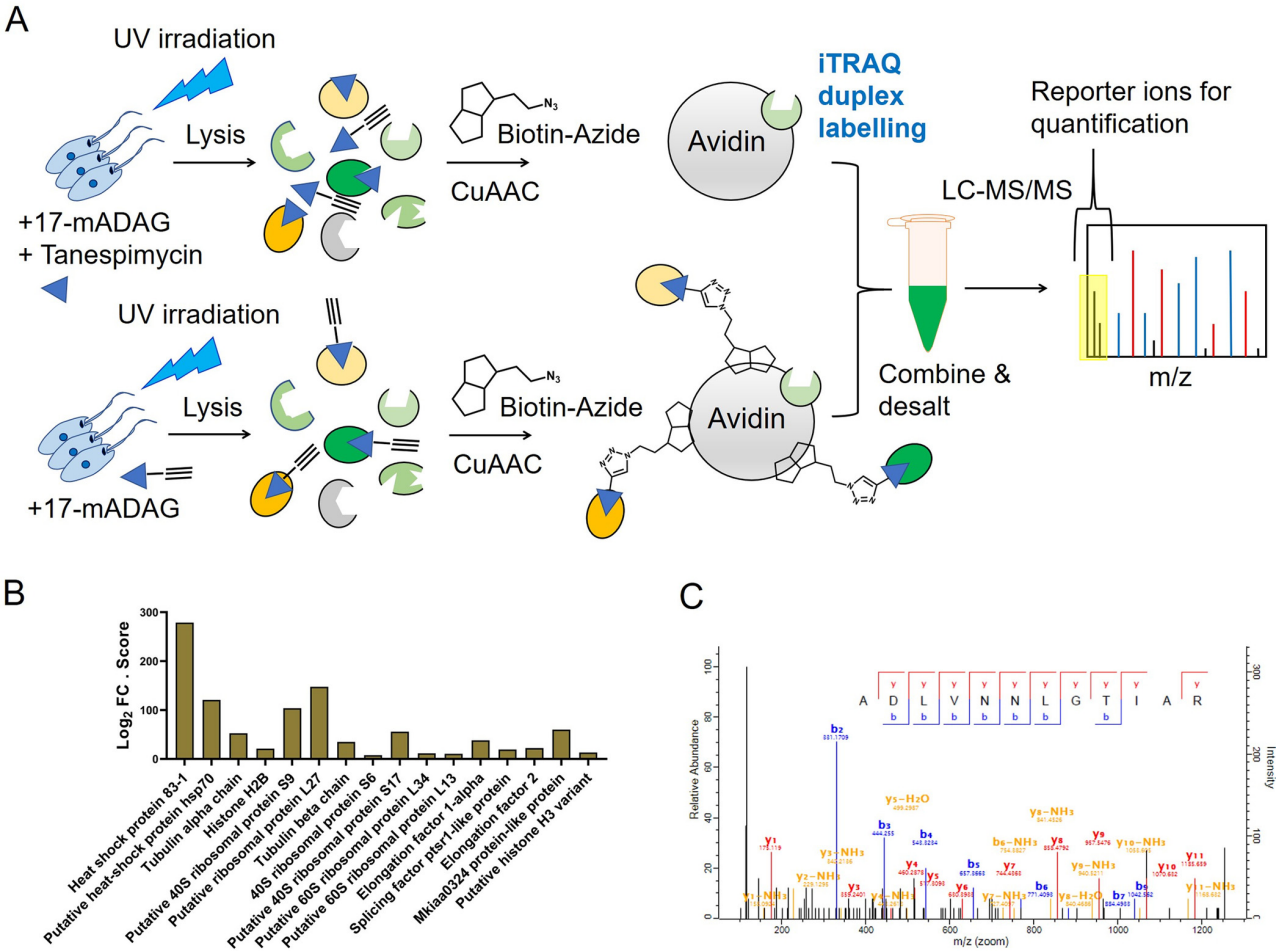
(A) Schematic of the competitive AfBPP using the probe (17-mADAG) and the competitor (tanespimycin) combined with iTRAQ duplex labeling-based quantitative proteomic MS in L. mexicana promastigotes. (B) Protein targets of 17-mADAG identified across three replicate AfBPP-iTRAQ-LC-MS/MS experiments presented in a bar chart. The fold change (FC) in abundance of the enriched proteins in the 17-mADAG-treated samples relative to that in the 17-mADAG plus the competitor (tanespimycin)-treated samples in log_2_ scale times the observed MaxQuant search score of each identified protein is shown. (C) A representative MS/MS spectrum of a tryptic peptide of L. mexicana Hsp83-1 identified by database search using MaxQuant showing the observed y and b ion fragmentation patterns.

For an unbiased validation of tanespimycin (Fig. 4A) target engagement in L. mexicana, we developed a quantitative chemical proteomics workflow. We first synthesized a novel tanespimycin probe 17-minimalist alkyne diazirine aminogeldanamycin (17-mADAG) (Fig. 4B) that closely mimics the chemical structure of tanespimycin but is equipped with a terminal alkyne tag for click chemistry and diazirine functionality (47) for photo-cross-linking of target proteins in proximity. The 17-mADAG probe was utilized in a live parasite competitive photoaffinity-based protein profiling (AfBPP) (33–35) coupled with iTRAQ quantitative proteomic MS (Fig. 5A). Prior to performing the chemical proteomics target validation, we evaluated the antileishmanial activity of the probe in L. mexicana log-phase promastigotes in comparison with that of tanespimycin. Both compounds exhibited potent and comparable activity, with 50% inhibition concentration (IC_50_) values of 211 nM and 640 nM for tanespimycin and 17-mADAG, respectively, in antiproliferative assays (see Fig. S4). Additionally, docking analyses, in agreement with a recent study (48), also revealed similar binding poses for 17-mADAG and tanespimycin at the ATP-binding pocket of L. mexicana Hsp83-1 (see Fig. S5), with both compounds exploiting similar binding interactions and solvent-exposed sidechains on the position C-17.

10.1128/mSystems.00089-21.4 FIG S4 Antileishmanial activity of tanespimycin (red) and 17-mADAG (blue) against L. mexicana promastigotes. Download FIG S4, PDF file, 0.1 MB. Copyright © 2021 Kalesh et al. 2021 Kalesh et al. https://creativecommons.org/licenses/by/4.0/ This content is distributed under the terms of the Creative Commons Attribution 4.0 International license.

10.1128/mSystems.00089-21.5 FIG S5 L. mexicana Hsp83-1 binding site with tanespimycin (green) and 17-mADAG (cyan) revealed by docking analysis showing solvent exposed C-17 side chains (top) and key interactions (bottom). Conventional hydrogen bonds (magenta), carbon-hydrogen bonds (red), hydrophobic interactions (yellow), and carbon-pi interactions (purple) are presented. Download FIG S5, PDF file, 0.4 MB. Copyright © 2021 Kalesh et al. 2021 Kalesh et al. https://creativecommons.org/licenses/by/4.0/ This content is distributed under the terms of the Creative Commons Attribution 4.0 International license.

Using equimolar concentrations of the probe and the competitor tanespimycin, probe-only-treated parasites as well as probe- and tanespimycin-treated parasites in three replicates were subjected to UV irradiation. Following cell lysis, the extracts were clicked with a biotin-azide, and the labeled proteins were affinity enriched on NeutrAvidin-agarose resin. After on-bead tryptic digestion, the samples were subjected to iTRAQ duplex labeling and analyzed by LC-MS/MS. A total of 16 target proteins (Fig. 5B; see also Table S4) were retained after filtering away proteins identified with fewer than 2 unique peptides, contaminants, and endogenous biotinylated proteins. While Hsp83-1 (see Fig. 5C for a representative LC-MS/MS spectrum of a tryptic peptide) pulled out by the probe revealed its on-target engagement, the enrichment of other proteins shows, for the first time, that Hsp90 inhibition targets multiple proteins in the *Leishmania* parasite.

10.1128/mSystems.00089-21.9 TABLE S4 Targets of 17-mADAG identified by competitive AfBPP followed by iTRAQ labeling-based quantitative proteomics. Download Table S4, XLSX file, 0.1 MB. Copyright © 2021 Kalesh et al. 2021 Kalesh et al. https://creativecommons.org/licenses/by/4.0/ This content is distributed under the terms of the Creative Commons Attribution 4.0 International license.

### Differential quantitative proteomic profiling of the direct effect of Hsp90 inhibition on the L. mexicana promastigote proteome.

**FIG 6:**
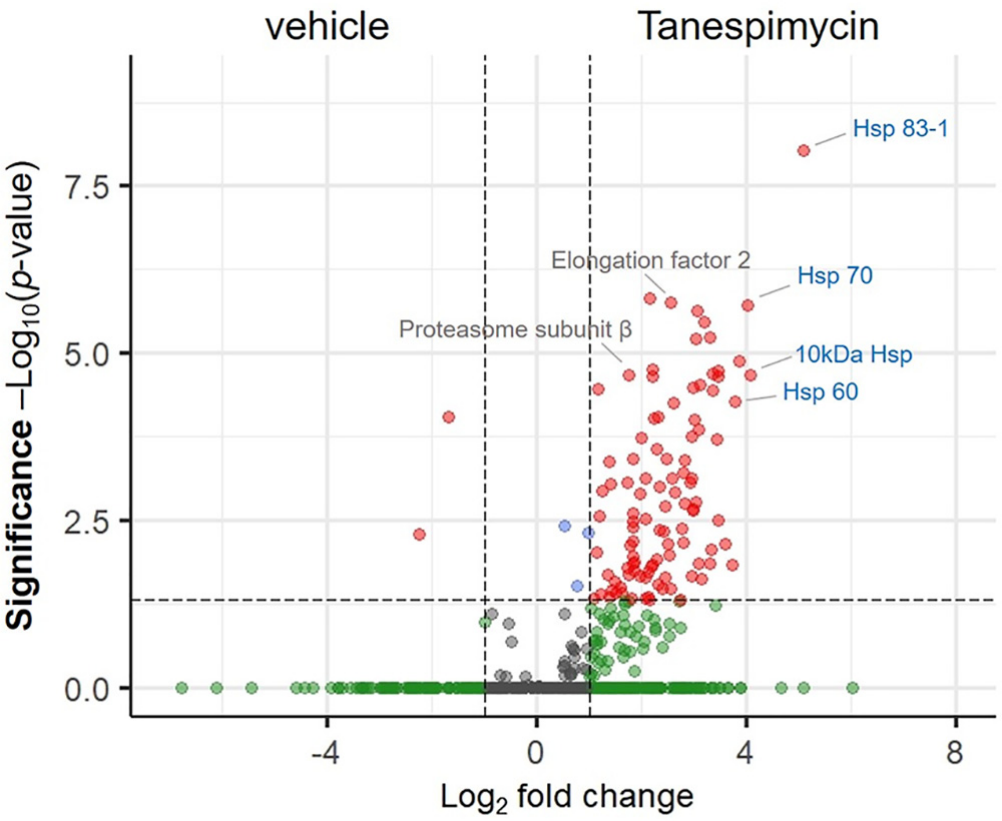
Volcano plot showing the enrichment of L. mexicana promastigote proteins upon Hsp90 inhibition with tanespimycin treatment (1 μM for 12 h) over that with vehicle (DMSO) treatment across three replicates profiled by label-free quantification (LFQ) proteomic MS. A modified *t* test with permutation-based FDR statistics was applied (250 permutations, FDR = 0.05) to compare tanespimycin-treated and vehicle-treated groups.

For additional characterization of the direct effect of Hsp90 inhibition on the L. mexicana promastigote proteome, we performed differential quantitative proteomic profiling using label-free quantification (LFQ) proteomic MS (49) of the inhibitor versus vehicle treatment conditions. The lysates after in-solution tryptic digestion and LC-MS/MS were analyzed by MaxLFQ (50). As in the case of the nascent proteins, the Hsp90 inhibitor affected the expression levels of hundreds of L. mexicana proteins (Fig. 6; see also Table S5). Similarly, in agreement with the NSP profiling, Hsp90 and several other chaperon proteins remained among the most significantly elevated proteins.

10.1128/mSystems.00089-21.10 TABLE S5 Quantitative proteomic profiling of direct effect of tanespimycin treatment (1 μM for 12 h) on L. mexicana proteome using label-free quantification (LFQ) proteomic MS. Download Table S5, XLSX file, 0.1 MB. Copyright © 2021 Kalesh et al. 2021 Kalesh et al. https://creativecommons.org/licenses/by/4.0/ This content is distributed under the terms of the Creative Commons Attribution 4.0 International license.

## DISCUSSION

*Leishmania* spp. parasites are among the most ancient lineages in the evolution of eukaryotes and possess a peculiar genomic organization with a notable absence of introns. The proteome and the transcriptome are poorly correlated in this organism (51). Quantitative proteomic MS that provides a direct measurement of the proteome is therefore particularly suitable for the measurement of translation in *Leishmania* spp. Here, we report the first systematic study of the effect of Hsp90 inhibition on global protein synthesis in L. mexicana parasites using BONCAT metabolic labeling and quantitative proteomic MS. This robust methodology enabled highly sensitive profiling of relative changes in the synthesis of hundreds of L. mexicana proteins as functions of the dose and duration of the Hsp90 inhibitor treatment. As a master regulator of protein homeostasis, it is conceivable that the inhibition of Hsp90 will have major consequences on the stabilization, activation, and degradation of many cellular proteins. In mammalian cells, molecular chaperones play dual roles of facilitating polypeptide elongation and cotranslational folding at the ribosome (41), and Hsp90 inhibition affects the protein synthesis capacity of the ribosome (42).

Our results provide the first protein-level evidence that severe Hsp90 inhibition in *Leishmania* spp. causes a decrease in the synthesis of many RPs, while the synthesis of many virulence factors and quality control proteins remains upregulated. Although RPs act as integral structural constituents of the ribosome, many of them are known to have extraribosomal functions. For instance, in prokaryotes, RPL4 is known to inhibit translation of polycistronic mRNAs (52). The eukaryotic RPS3, a component of the 40S ribosomal small subunit, acts as a DNA repair endonuclease (53). It also regulates cell growth and apoptosis in some cell lines (54). In human cell lines, Hsp90 directly interacts with RPS3, and this interaction has been reported to protect the RP from ubiquitination and subsequent proteasome-dependent degradation, thereby retaining the function and biosynthesis of the ribosome (42). In eukaryotes, many RPs regulate the synthesis of the protein and RNA components of the ribosome itself (55). RPs S13, S14, L12, and L30 can inhibit their own mRNA splicing (56–59). Similarly, RPs S28 and L2 are known to shorten their own mRNA half-lives (60, 61). In *Drosophila*, RPL22 associates with the liker histone H1 and causes repression of several genes (62). In yeast, RP L6 positively modulates RNA polymerase III transcription (63). In L. donovani, 60S ribosomal L23a has been found to be overexpressed in different sodium antimony gluconate-resistant strains and play crucial roles in parasite survival and proliferation (64). The cytosolic ribosome was recently identified as the primary drug target of the aminoglycoside antileishmanial drug paromomycin (65). The statistically significant preferential downregulation of many RPs upon treatment with the Hsp90 inhibitor revealed in our study indicate that the major downstream effect of the inhibitor in L. mexicana is on the parasite ribosome.

SOD, an increasingly expressed protein upon Hsp90 inhibition, is an important component of *Leishmania*’s antioxidant system and is a known virulence factor (44). The dismutation reaction catalyzed by SOD generates hydrogen peroxide within mitochondria. This has been shown to play a crucial role in the differentiation of Leishmania amazonensis promastigotes to infective amastigotes (44). Prolonged treatment with Hsp90 inhibitors also induces promastigote-to-amastigote differentiation in *Leishmania* spp. (6). However, the downstream molecular mechanisms of this differentiation process remain unknown. It is likely that the increased expression of SOD partly contributes to the Hsp90 inhibition-mediated differentiation in *Leishmania* spp. Among the other preferentially synthesized proteins, malate dehydrogenase, an important enzyme in the tricarboxylic acid cycle, was previously identified as a trigger for polyclonal B-cell activation during the acute phase of Trypanosoma cruzi infection, facilitating the infection *in vivo* (66). Similarly, sucrose-phosphate synthase-like protein, an enzyme involved in sugar metabolism, was previously identified among proteins that are highly expressed in a virulent strain of L. major but not in an avirulent strain of the same species (67). The increased synthesis of histone H4, which is in agreement with a recent ribosome profiling study in L. donovani (14), indicates the possibility of chromatin reorganization in *Leishmania* under cellular stress caused by Hsp90 inhibition (68, 69). The preferentially upregulated proteins identified in this study, many of which are identified or presumed virulence factors, represent a set of target proteins for systematic development of drug combination studies with the Hsp90 inhibitor treatment. Our results thus provide a rich resource for further studies on the prospects of Hsp90 inhibition in the *Leishmania* parasite.

We also report a systematic chemical proteomics target validation of tanespimycin in L. mexicana. The combination of competitive activity/affinity-based protein profiling (ABPP/AfBPP) with quantitative proteomic MS has evolved as a powerful unbiased method for target and off-target profiling of biologically active agents (33–35). Our chemical proteomics target profiling using the novel tanespimycin analogue, the minimalist terminal alkyne diazirine compound 17-mADAG, revealed that the classical Hsp90 inhibitor not only engages with its presumed target Hsp90 in L. mexicana but also affects multiple proteins involved in protein synthesis and quality control. Hsp90 directly associates with many clients and forms multiprotein complexes (1). Therefore, it is possible that some of the identified off-targets were pulled down due to their close physical association with the main target Hsp90, as the photo-cross-linking is proximity driven (70). The identification of translation elongation factors and RPs in the chemical proteomics experiment provides additional validation for the BONCAT-iTRAQ quantitative proteomic MS findings that Hsp90 inhibition affects protein translation and RP synthesis in L. mexicana.

## MATERIALS AND METHODS

### *Leishmania* cell culture.

L. mexicana strain M379 (MNYC/BC/62/M379) promastigotes were grown in T-25 flasks at 26°C in Schneider’s insect medium (Sigma-Aldrich) supplemented with 0.4 g/liter NaHCO_3_, 0.6 g/liter anhydrous CaCl_2_, and 10% fetal bovine serum (FBS) (pH 7.2).

### Growth inhibition assay.

Growth inhibition assays were performed as previously described (71). Briefly, serial 3-fold dilutions of the two compounds (tanespimycin and 17-mADAG) in triplicates in the growth medium (Schneider’s insect medium, pH 7.2 supplemented with 0.4 g/liter NaHCO_3_, 0.6 g/liter anhydrous CaCl_2_, and 10% FBS) starting with a 50 μM concentration were performed in flat-bottom 96-well cell culture microtiter plates. Amphotericin B was used as positive control, and DMSO was used as negative control. L. mexicana promastigotes were plated at a concentration of 10^6^ parasites/ml. After 48 h of incubation, the parasite viability was determined by alamarBlue assay (20 μl/well freshly prepared 0.15 mg/ml resazurin in phosphate-buffered saline [PBS]), reading the fluorescence emission at 590 nm (bandwidth, 10 nm) following excitation at 550 nm (bandwidth, 10 nm) using a BioTek SYNERGY HTX microplate reader.

### *In situ* photoaffinity labeling.

L. mexicana promastigotes grown to mid-log phase (∼5 × 10^6^ parasites/ml) in Schneider’s insect medium supplemented with 10% FBS were plated in 24-well plates with 500 μl medium per well. 17-mADAG was added to the culture medium with or without equimolar amounts of tanespimycin for different durations (4 h to 16 h), after which, the medium was replaced with PBS and the parasites were irradiated with UV light (6-W lamp, ∼365-nm wavelength) for 10 min on ice. The parasites with or without UV-irradiation were collected by centrifugation at 1,000 × *g* for 3 min, washed with PBS, and immediately lysed using a lysis buffer (50 mM HEPES, pH 7.4, 150 mM NaCl, 4% SDS, 250 U Benzonase). Protein concentrations of the lysates were determined using a Bio-Rad DC protein assay, and lysates at a 1-mg/ml concentration were subjected to click chemistry with biotin-azide. Following the click reactions, proteins were precipitated using a methanol-chloroform-water mixture (4:1:3), washed, resolubilized in a buffer with 4% SDS, diluted to 0.1% SDS, and subjected to affinity enrichment on NeutrAvidin-agarose resin.

### Tanespimycin treatment and metabolic labeling of nascent proteins.

L. mexicana promastigotes grown to mid-log phase (∼5 × 10^6^ parasites/ml) in Schneider’s insect medium were placed in methionine-free Schneider’s medium supplemented with 10% dialyzed FBS and 50 μM azidohomoalanine (AHA). For profiling the effects of tanespimycin treatment on the nascent protein synthesis by iTRAQ 4-plex labeling, the parasites in three replicates were treated with three different concentrations (2, 10, and 25 μM) of the Hsp90 inhibitor for 2 h. DMSO instead of tanespimycin was used as a vehicle control. For profiling the temporal effects of tanespimycin treatment by iTRAQ duplex experiments, a fixed 50 μM concentration of tanespimycin was used in three replicates for 1-h and 4-h durations along with equal durations of vehicle (DMSO) treatments. Following treatments, the parasites were lysed using lysis buffer (50 mM HEPES, pH 7.4, 150 mM NaCl, 4% SDS, 250 U Benzonase), and the protein concentrations were determined using a Bio-Rad DC protein assay.

### Click chemistry and affinity enrichment.

Whole-cell extracts of the *Leishmania* parasites at a 1-mg/ml concentration were treated with freshly premixed click chemistry reaction cocktail (100 μM capture reagent [biotin-azide for 17-mADAG treatment or biotin-alkyne for the nascent protein profiling; 10 mM stock solutions in DMSO], 1 mM CuSO_4_ solution [50 mM stock solution in Milli-Q water], 1 mM tris[2-carboxyethyl]phosphine solution [TCEP solution; 50 mM stock solution in Milli-Q water], and 100 μM tris[{1-benzyl-4-triazolyl}methyl]amine solution [TBTA solution; 10 mM stock solution in DMSO]) for 3 h at room temperature. Proteins were precipitated by adding methanol (4 volumes), chloroform (1.5 volumes), and water (3 volumes) and collected by centrifugation at 14,000 × *g* for 5 min. The protein precipitates were washed twice with methanol (10 volumes; centrifugation at 14,000 × *g* for 5 min to collect the pellets), and the supernatants were discarded. The protein pellets were air dried at room temperature for 20 min and stored in a −80°C freezer.

For affinity enrichment, the air-dried protein pellets obtained after the click reactions and protein precipitation were dissolved in phosphate-buffered saline (PBS) with 2% SDS to a 5-mg/ml concentration by sonication. In a typical affinity enrichment experiment, 300 μg of the parasite lysate subjected to click reaction and protein precipitation was resuspended in 50 μl 2% SDS in PBS. The samples were then diluted 20-fold with PBS so that the final SDS amount was 0.1%. The samples were centrifuged at 10,000 × *g* for 5 min to remove insoluble debris, and the clear soluble portion was used for the affinity enrichment. Typically, 30 μl of NeutrAvidin-agarose beads, freshly washed three times with 0.1% SDS buffer (0.1% SDS in PBS), was added to each of the samples, and the mixtures were rotated on an end-over-end rotating shaker for 1.5 h at room temperature. The beads were then washed 3 times with 1% SDS in PBS, 3 times with 6 M urea in PBS, 3 times with PBS, and once with 25 mM triethylammonium bicarbonate (TEAB) buffer. Each washing was performed with 20 volumes of the washing solutions with respect to the bead volume, and centrifugation of the beads between washing steps was carried out at 2,000 × *g* for 1 min at room temperature.

### On-bead reduction, alkylation, and tryptic digestion.

Thoroughly washed beads from the affinity enrichment step were resuspended in 150 μl of 25 mM TEAB buffer and treated with 10 mM TCEP (200 mM stock solution in water) for 45 min at 35°C. The beads were washed once with 25 mM TEAB buffer, resuspended in 150 μl of 25 mM TEAB buffer, and treated with 15 mM chloroacetamide (CAA; 200 mM stock solution in water) in the dark for 20 min at room temperature. The beads were again washed with 25 mM TEAB buffer, resuspended in 200 μl of fresh 50 mM TEAB buffer, and treated with 5 μg of sequencing-grade modified trypsin at 37°C for 16 h. The samples were centrifuged at 5,000 × *g* for 5 min at room temperature to collect the supernatant. The beads were washed twice with 50% (vol/vol) acetonitrile (ACN) containing 0.1% (vol/vol) formic acid (FA; 50 μl for each wash) and mixed with the previous supernatant. The collected tryptic peptides were acidified to pH 3 using FA and evaporated to dryness. The peptides were then redissolved in 0.1% (vol/vol) FA solution in water and subjected to desalting on Pierce C_18_ spin columns (Thermo Scientific; catalog number 89873) according to the manufacturer’s instructions. The peptides were evaporated to complete dryness under a vacuum.

### iTRAQ labeling.

The iTRAQ labeling reactions were carried out as previously described (36). Briefly, the dried peptides were resuspended in equal volumes (30 μl) of dissolution buffer (0.5 M TEAB buffer supplied with the iTRAQ reagents multiplex kit). Two hundred ten microliters of absolute ethanol was added to each iTRAQ reagent vial preequilibrated to room temperature. The contents of each iTRAQ reagent vial were split into 3 equal portions (70 μl each), and each portion was quickly transferred to the respective vials of desalted and dried peptide digests. The labeling reactions were performed for 1.5 h at 25°C and quenched with 100 mM Tris base solution (1 M stock solution). The samples labeled with the different iTRAQ channels from the same experiments were pooled into a fresh vial and concentrated on a speed-vac. The dried peptides were reconstituted in water with 0.1% (vol/vol) FA and 2% (vol/vol) ACN and subjected to desalting on C-18 Sep-Pak Classic cartridges (Waters; WAT051910) following manufacturer’s instructions. The eluted peptides were concentrated on a speed-vac and subjected to a second round of cleaning up on HILIC TopTip (PolyLC; TT200HIL) solid-phase extraction tips following manufacturer’s instructions. The eluted peptides were concentrated on a SpeedVac and reconstituted in aqueous 0.1% (vol/vol) FA.

### Tanespimycin treatment and in-solution tryptic digestion.

L. mexicana promastigotes grown to mid-log phase (∼5 × 10^6^ parasites/ml) in hemoflagellate-modified minimum essential medium (HOMEM) supplemented with 10% FBS were treated in three replicates with tanespimycin (1 μM) or DMSO for 12 h. Following treatments, the parasites were lysed using lysis buffer (20 mM Tris-HCl, pH 8.5, 8 M urea, 1 mM dithiothreitol [DTT], Roche cOmplete EDTA-free protease inhibitor cocktail), passed through a 29-gauge needle, and centrifuged at 4°C for 5 min at 5,000 × *g*, and the protein concentrations in the clear lysates were determined. Three hundred micrograms of each lysate was subjected to in-solution tryptic digestion using 6 μg of sequencing-grade modified trypsin at 37°C for 16 h after reduction with 5 mM DTT and *S*-carbamidomethylation with 10 mM iodoacetamide. The samples were acidified to pH 3 using FA, desalted on C_18_ Sep-Pak Classic cartridges, concentrated on a SpeedVac, and reconstituted in aqueous 0.1% (vol/vol) FA.

### LC-MS/MS analysis.

The iTRAQ-labeled peptides and in-solution tryptic digests were resolved on an ekspert nanoLC 425 with a low microgradient flow module (Eksigent) using a YMC-Triart C_18_ column (12 nm, S-3 μm, 150 mm by 0.3 mm inside diameter [i.d.], 1/32 in.; part number TA12S03-15H0RU). A C_18_ trap column (Trap-YMC-Triart 12 nm S-5 μm, 5 mm by 0.5-mm i.d., 1/32 in.; part number: TA12S05-E5J0RU) was connected prior to the main separating column. Five microliters of iTRAQ-labeled peptides was separated by mobile phase A (0.1% FA in water) and mobile phase B (0.1% FA in ACN) at a flow rate of 5 μl/min over 87 min. The gradient used was the following: 3% B to 5% B (0 to 2 min), 5% B to 30% B (2 to 68 min), 30% B to 35% B (68 to 73 min), 35% B to 80% B (73 to 75 min), 80% B (75 to 78 min), 80% B to 3% B (78 to 79 min), and 3% B (79 to 87 min). For the in-solution tryptic digests, a 90-min total gradient with 2-min postrun equilibration in ending buffer was performed. Five microliters of sample peptides was separated by mobile phase A and mobile phase B. The gradient used for the in-solution tryptic digests was the following: 3% B to 30% B (0 to 60 min), 30% B to 40% B (60 to 77 min), 40% B to 80% B (77 to 79 min), 80% B (79 to 82 min), 80% B to 3% B (82 to 84 min), and 3% B (84 to 90 min). The MS analyses were performed on a TripleTOF 6600 system (Sciex) in high-resolution mode. The MS acquisition time was set from gradient time zero to 85 min, and the MS1 spectra were collected in the mass range of 400 to 1,500 *m/z* with 250-ms accumulation time per spectrum. Further fragmentation of each MS1 spectrum occurred with a maximum of 30 precursors per cycle and 33-ms minimum accumulation time for each precursor across the range of 100 to 1,500 *m/z* with ion selection +2 to +5, 500 cps intensity threshold and dynamic exclusion for 15 s. The MS/MS spectra were acquired in high-sensitivity mode.

### Proteomics MS data processing.

For protein identification and quantification, the .wiff files from the Sciex TripleTOF 6600 system were imported into MaxQuant (version 1.6.3.4) (72) with integrated Andromeda database search engine (73). The MS/MS spectra were queried against L. mexicana sequences from UniProt KB (8,524 sequences). The database search employed the following parameters: reporter ion MS2 with multiplicity 4plex for the iTRAQ 4-plex experiments and multiplicity 2plex for the iTRAQ duplex experiments, trypsin digestion with maximum 2 missed cleavages, carbamidomethylation of cysteine as fixed modification, oxidation of methionine and acetylation of protein N termini as variable modifications, maximum number of modifications per peptide set at 5, minimum peptide length of 6, and protein false discovery rate (FDR) of 0.01. Appropriate correction factors for the individual iTRAQ channels for both peptide N-terminal labeling and lysine side chain labeling as per the iTRAQ reagent multiplex kit were also configured into the database search. Label-free quantification in MaxQuant was performed using the built-in MaxLFQ algorithm. The type of LC-MS run was set as standard with multiplicity 1 for the LFQ experiments. Tanespimycin treatments and DMSO treatments were set as two separate parameter groups, and the MaxLFQ algorithm was applied independently to the two different parameter groups. The proteinGroups.txt file from the MaxQuant search output was processed using Perseus software (version 1.6.2.3) (74). Potential contaminants, reverse sequences, sequences only identified by site, and endogenous biotinylated proteins were filtered out. Additionally, proteins with fewer than 2 unique peptides identified were discarded. For each identified protein, the ratios of the tanespimycin treated and AHA-labeled reporter intensity corrected values to the vehicle treated and AHA-labeled reporter intensity corrected values or the ratios of the probe treated to probe and tanespimycin treated reporter intensity corrected values from the corresponding experiment were calculated yielding the fold change (FC). The FCs obtained for each protein were transformed into log_2_ scale, and volcano plots were generated following a *t* test on the three replicates, with significant data points determined with a permutation-based FDR calculation (FDR = 0.05, number of randomizations = 250).

### Gene ontology analysis.

The gene ontology terms (biological process and molecular function) significantly enriched in the nascent proteins synthesized under Hsp90 inhibition in L. mexicana promastigotes relative to the predicted whole proteome of the organism were derived using TriTrypDB (75). REVIGO software (76) was used to refine and visualize the enriched gene ontology terms.

### Protein-protein interaction network analysis.

Network analysis of the Hsp90 inhibitor-affected L. mexicana nascent proteome was performed by using the publicly available STRING database (version 11.0) of L. major strain Friedlin. The open source software platform Cytoscape (version 3.8.2) (77) was used for refining and visualizing the protein interaction network. Upregulated and downregulated proteins were presented as green and yellow nodes, respectively, and the STRING database score of each interaction was embedded in the width of the network edges.

### Homology modeling and docking.

The N-terminal domain of Hsp90 protein from L. major (PDB identifier [ID] 3Q5L) was used as a template to build the homology model of Hsp83-1 protein of L. mexicana. Both proteins share 96% sequence similarity. The fully automated homology modeling server Swiss-Model (78) was used to construct the model with default settings. The “protein preparation wizard” (79) implemented in Maestro 11.9 graphical user interface of the Schrodinger software suite (release 2019-1) was used to prepare the protein model. The bond type and bond orders were corrected. The hydrogen atoms were reassigned after deleting the original ones. The protonation states of acidic/basic amino acids were adjusted for pH 7.0. The OPLS2003e forcefield (80–82) was used for the restrained minimization of the protein with the convergence criterion of root mean square deviation (RMSD) of 0.3 Å for the heavy atoms. Ligands were sketched within Maestro and prepared using the LigPrep program of the Schrodinger software suite. The Epik 4.7 program (83, 84) was used to generate energetically accessible protonation states. The absolute stereochemistry of all the stereocenters was predefined, and no tautomeric forms were generated for the ligands. Molecular docking of the prepared ligands (17-AAG and GMD-probe) was performed using Autodock Vina (85, 86), implemented in the open source PyRx software (87) (version 0.8). The active-site residues were enclosed in a grid box cantered around the *x*, *y*, and *z* coordinates of −62.421, −28.580, and −32.474, respectively. The dimensions of the grid box were set to 25.43, 18.70, and 16.28 Å to include all the active-site residues. The exhaustiveness was set to 8, and all other default settings for Autodock Vina were used for the docking. The docked poses of ligands with the highest predicted binding affinity were reported, and images were generated using Discovery studio visualizer version 20.1.0 of Biovia Dassault Systèmes.

### Data availability.

All raw mass spectrometry proteomic data have been deposited to the ProteomeXchange Consortium via the PRIDE partner repository with the data set identifiers PXD022708 and PXD024764.
