# Supplementary material for: Quantitative Proteomics Reveals that Hsp90 Inhibition Dynamically Regulates Global Protein Synthesis in Leishmania mexicana
Source: mSystems. 2021 May 11;6(3):e00089-21. doi: 10.1128/mSystems.00089-21 (PMC8125071; doi:10.1128/mSystems.00089-21)
Supplement: FIG S1 [file mSystems.00089-21-sf001.pdf]

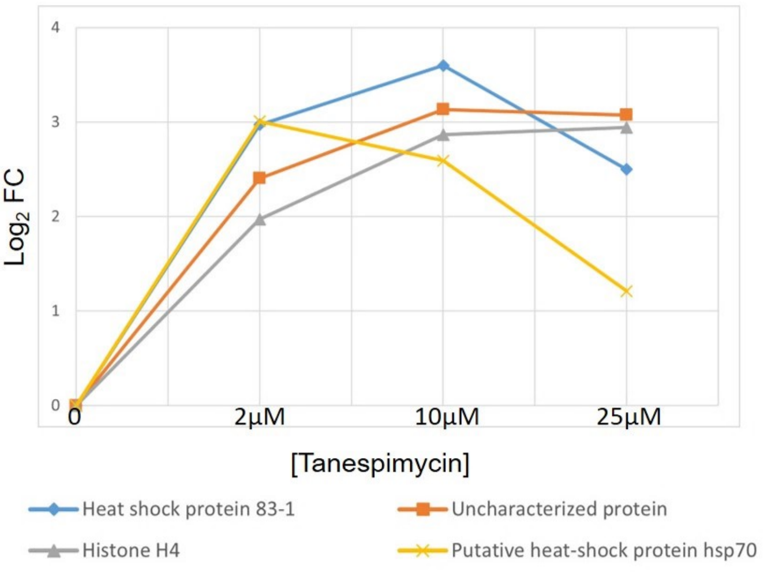
4
3
2
1
0
Log_2 FC
0
2μM
10μM
25μM
[Tanespimycin]
Heat shock protein 83-1
Uncharacterized protein
Histone H4
Putative heat-shock protein hsp70

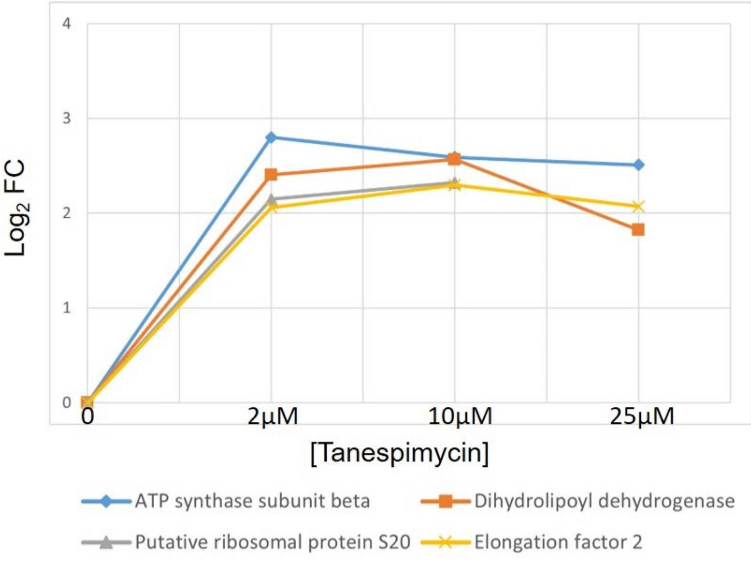
4
3
2
1
0
Log_2 FC
0
2μM
10μM
25μM
[Tanespimycin]
ATP synthase subunit beta
Dihydrolipoyl dehydrogenase
Putative ribosomal protein S20
Elongation factor 2

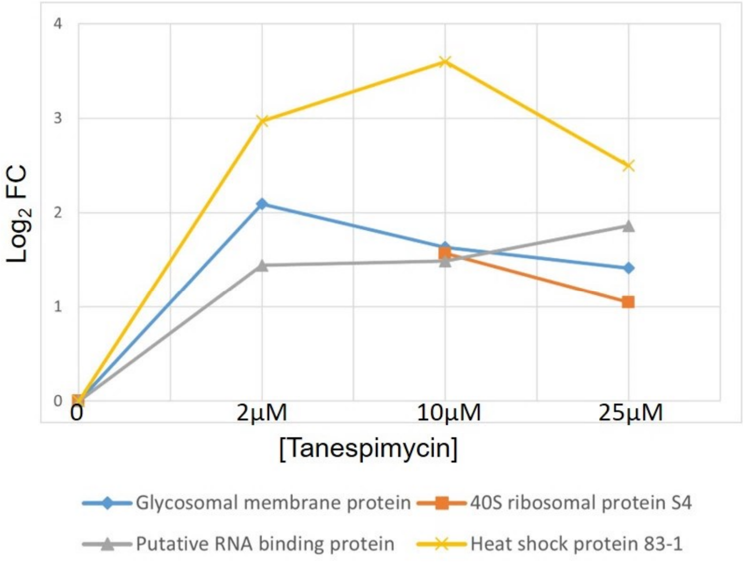
4
3
2
1
0
Log_2 FC
0
2μM
10μM
25μM
[Tanespimycin]
Glycosomal membrane protein
40S ribosomal protein S4
Putative RNA binding protein
Heat shock protein 83-1

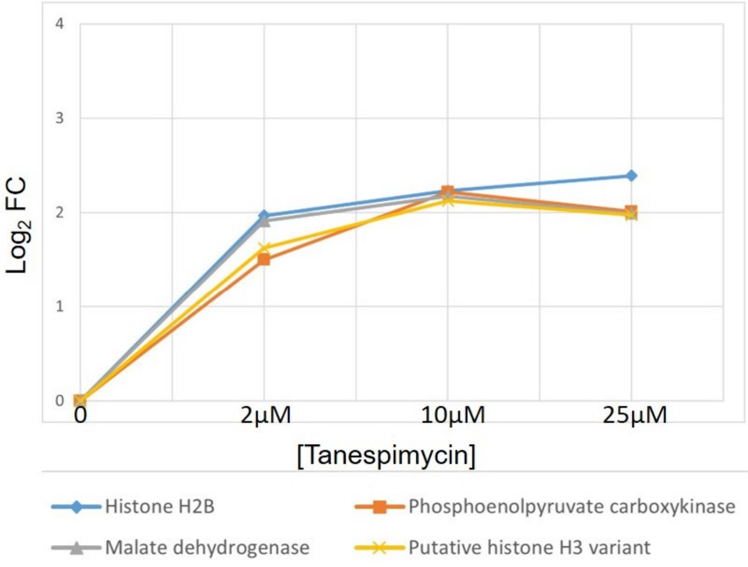
4
3
2
1
0
Log_2 FC
0
2μM
10μM
25μM
[Tanespimycin]
Histone H2B
Phosphoenolpyruvate carboxykinase
Malate dehydrogenase
Putative histone H3 variant

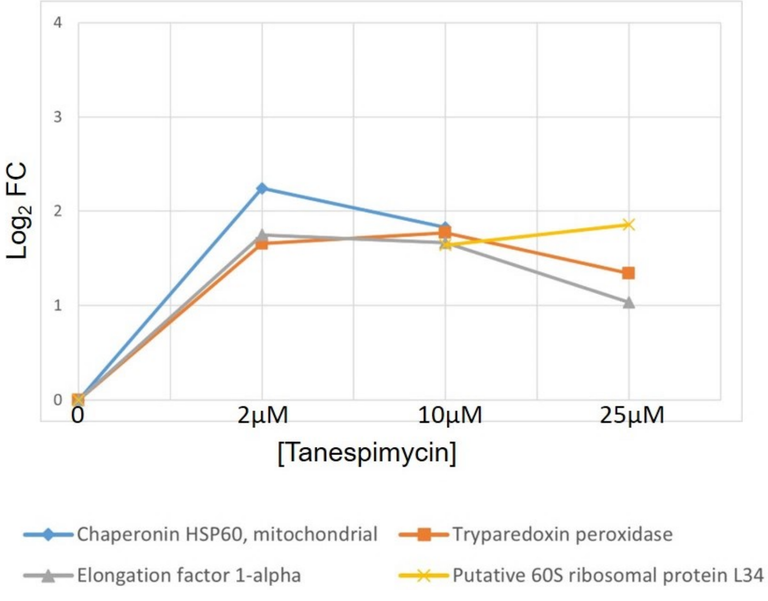
4
3
2
1
0
Log_2 FC
0
2μM
10μM
25μM
[Tanespimycin]
Chaperonin HSP60, mitochondrial
Tryparedoxin peroxidase
Elongation factor 1-alpha
Putative 60S ribosomal protein L34

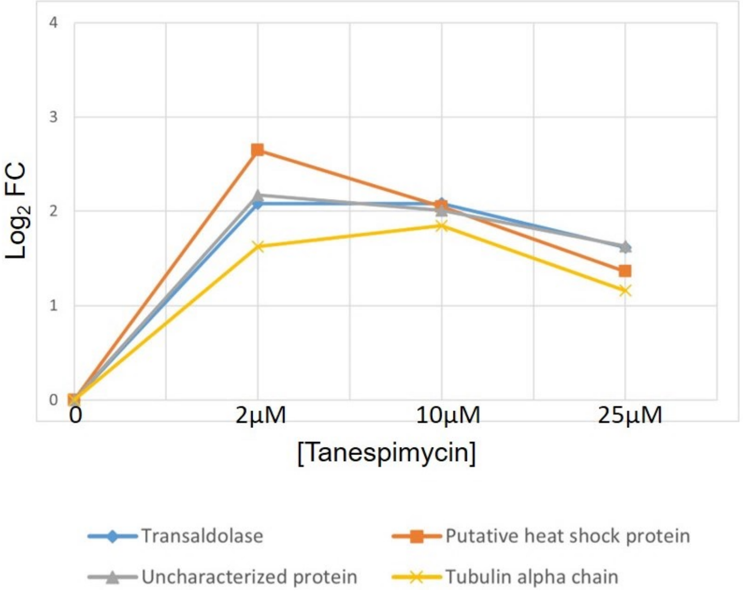
4
3
2
1
0
Log_2 FC
0
2μM
10μM
25μM
[Tanespimycin]
Transaldolase
Putative heat shock protein
Uncharacterized protein
Tubulin alpha chain
