# Supplementary figures and images for: Quantitative Proteomics Reveals that Hsp90 Inhibition Dynamically Regulates Global Protein Synthesis in Leishmania mexicana
Source: mSystems. 2021 May 11;6(3):e00089-21. doi: 10.1128/mSystems.00089-21 (PMC8125071; doi:10.1128/mSystems.00089-21)

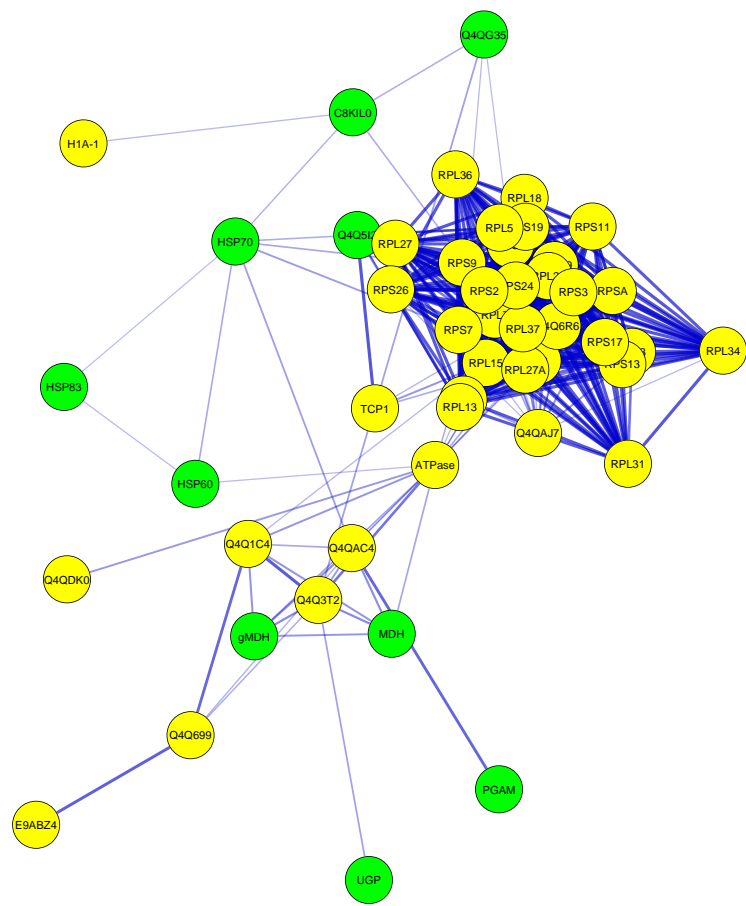

Supplement: FIG S2 [file mSystems.00089-21-sf002.pdf]

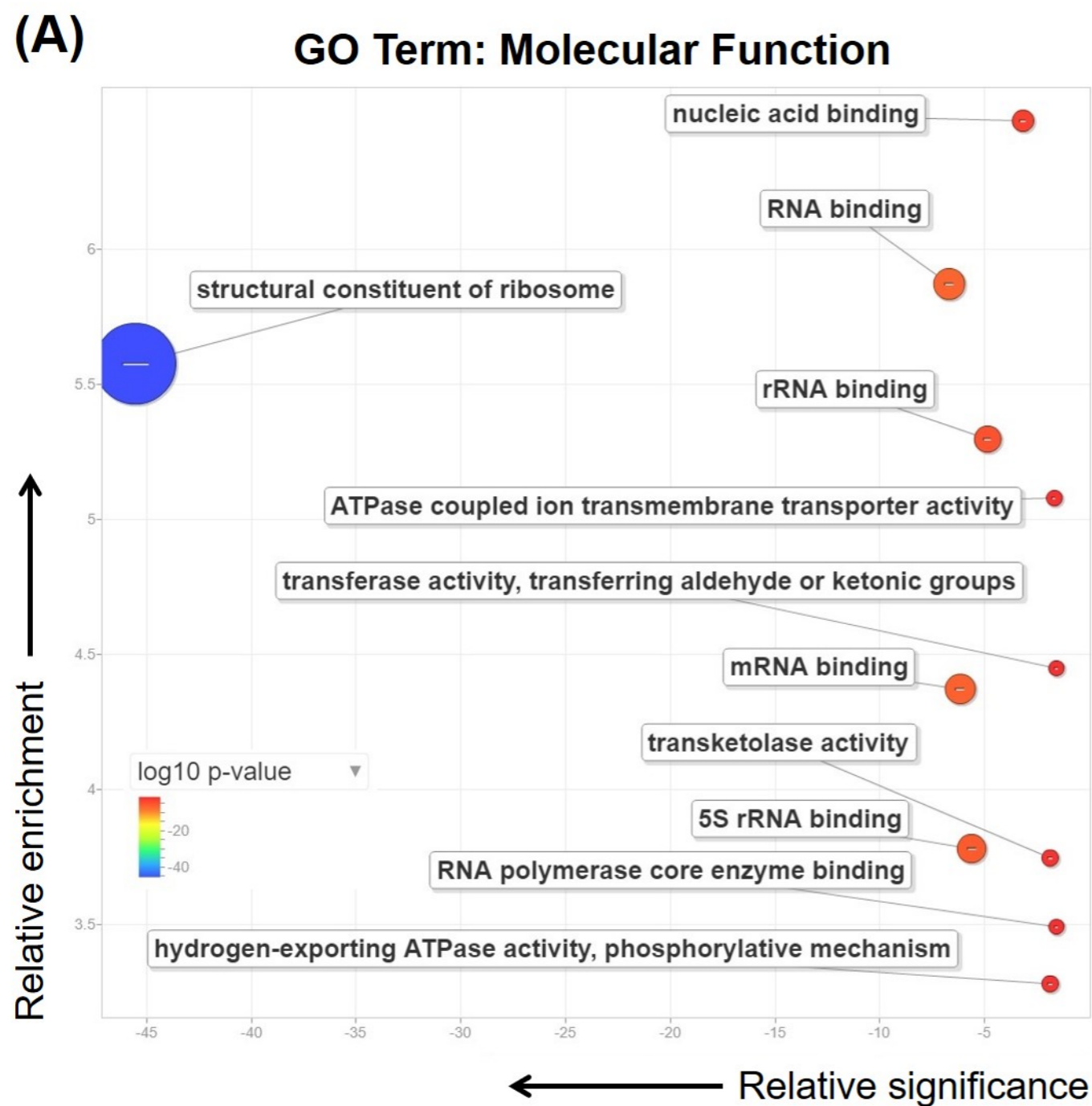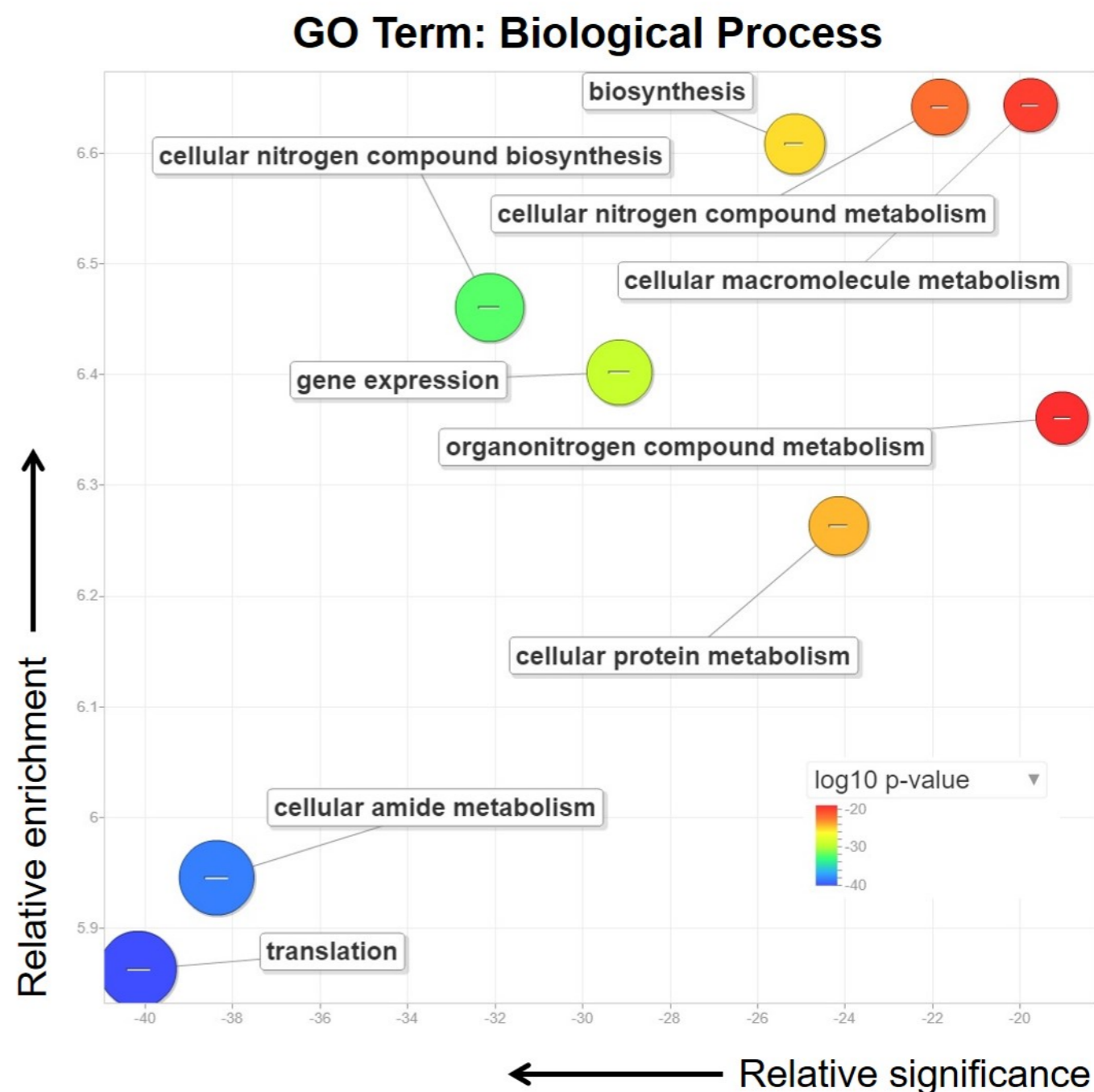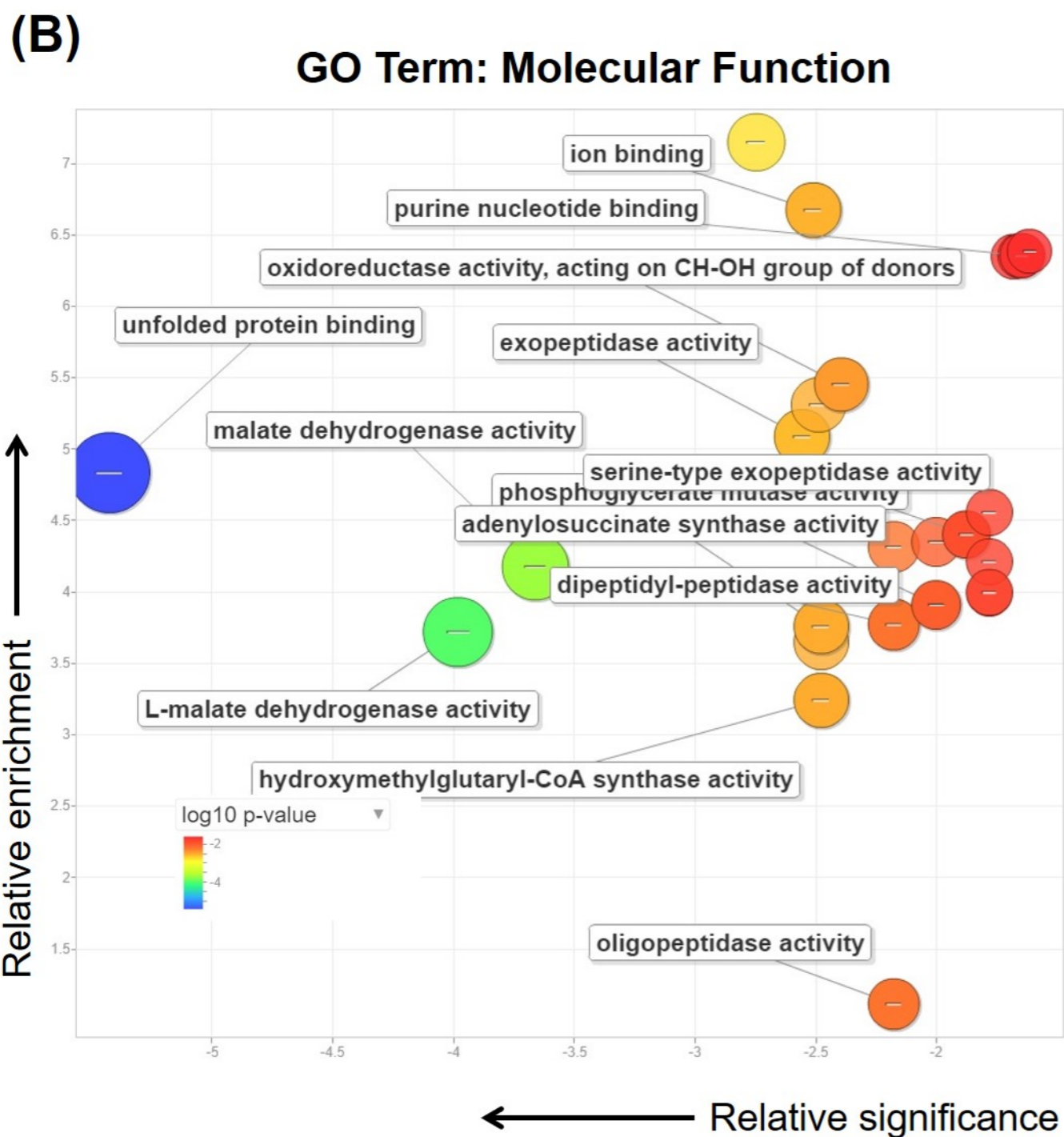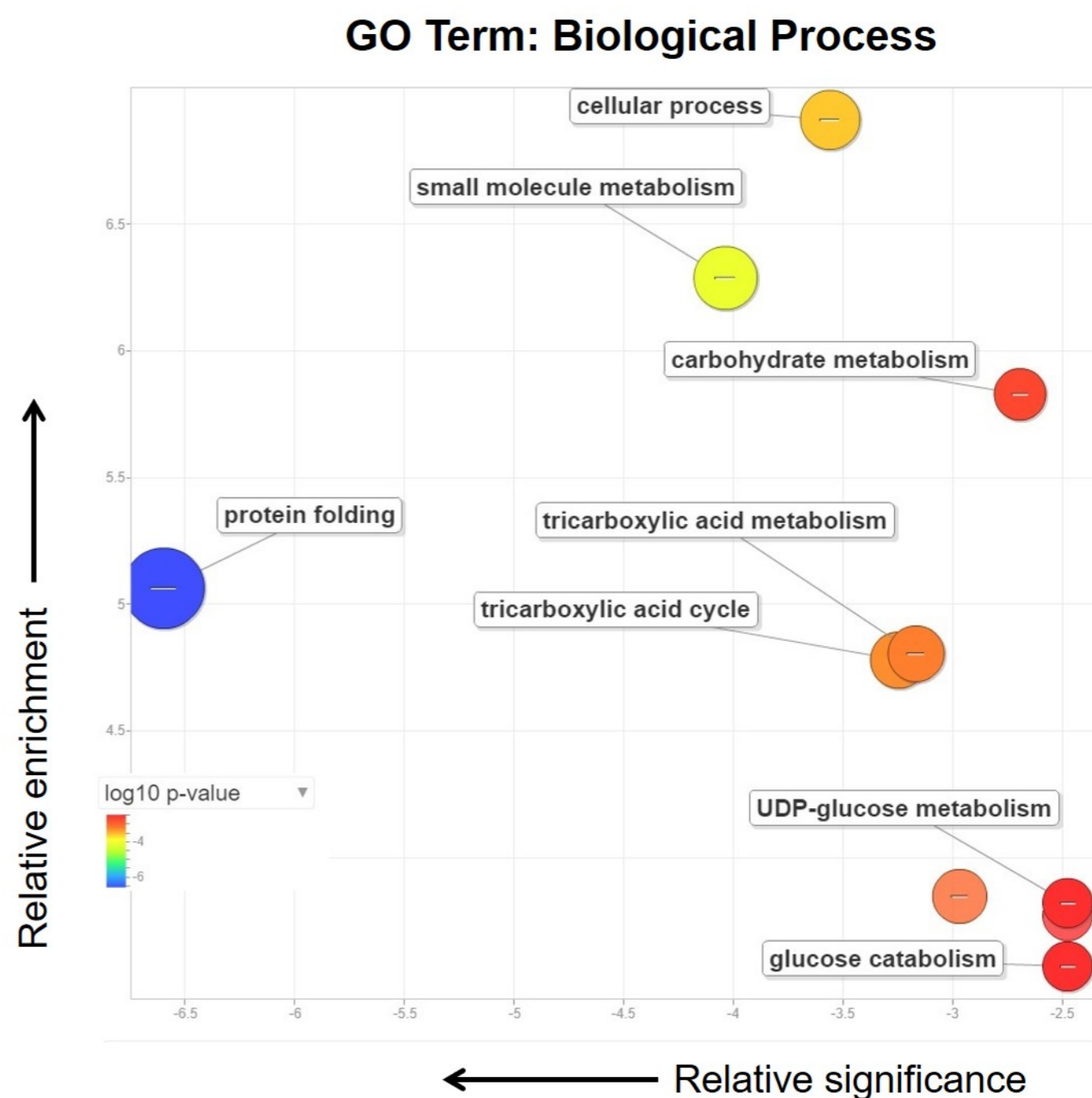

Supplement: FIG S3 [file mSystems.00089-21-sf003.pdf]

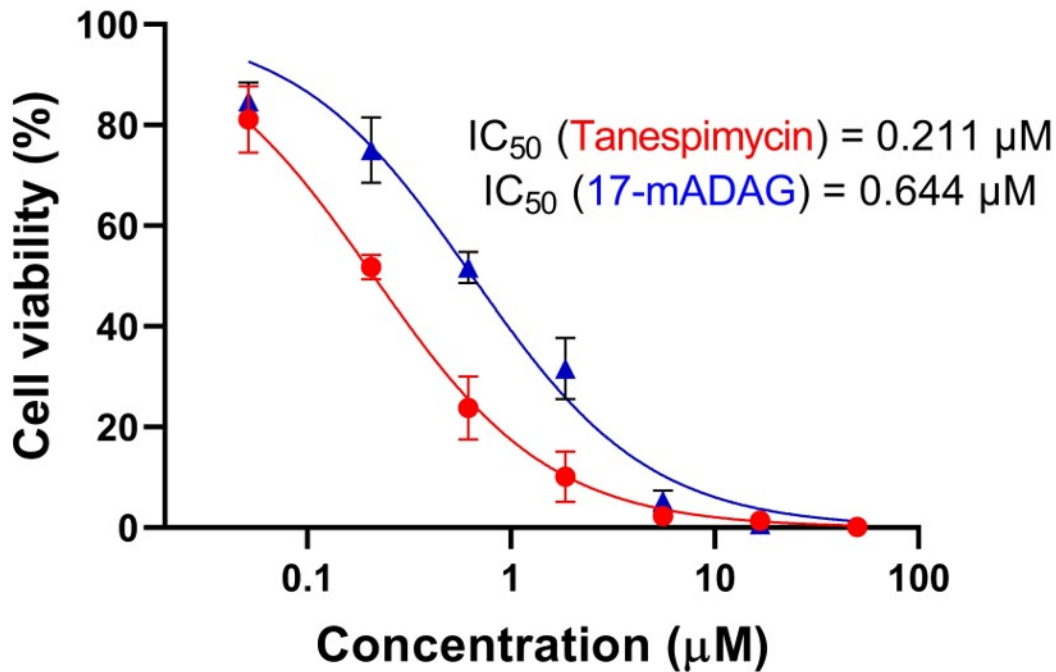

Supplement: FIG S4 [file mSystems.00089-21-sf004.pdf]

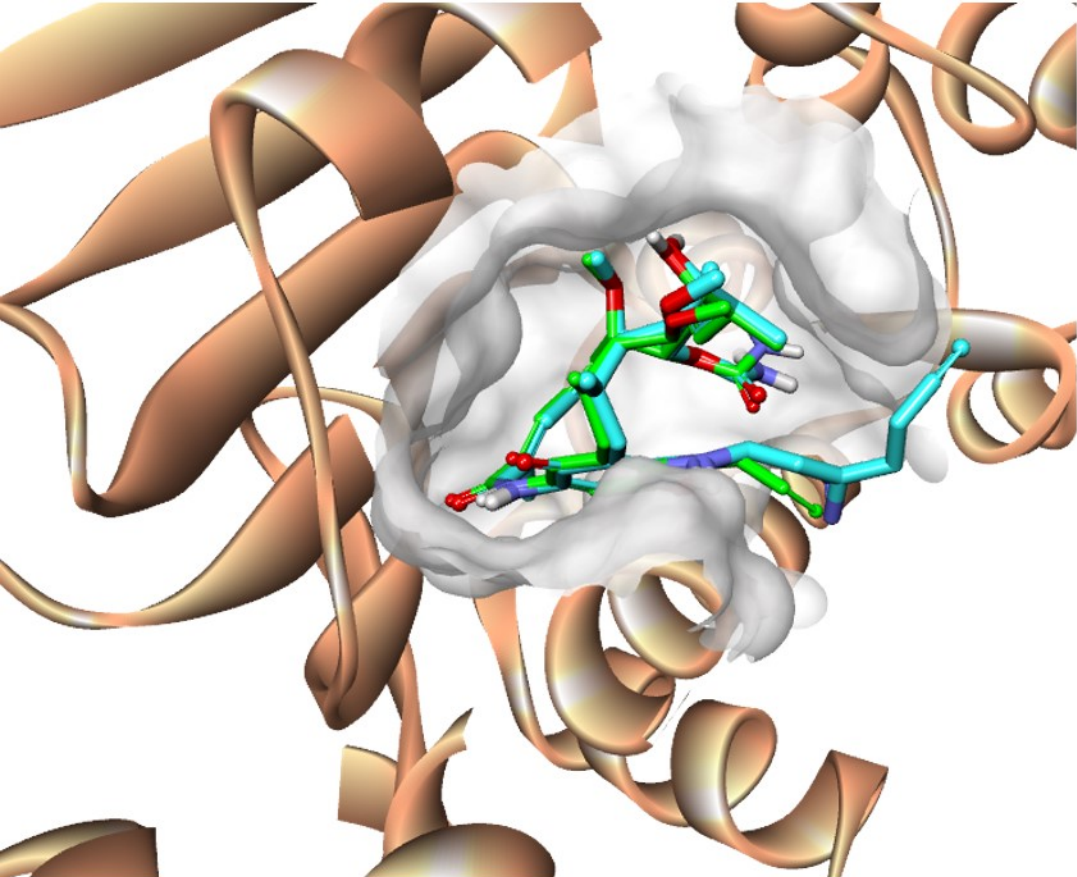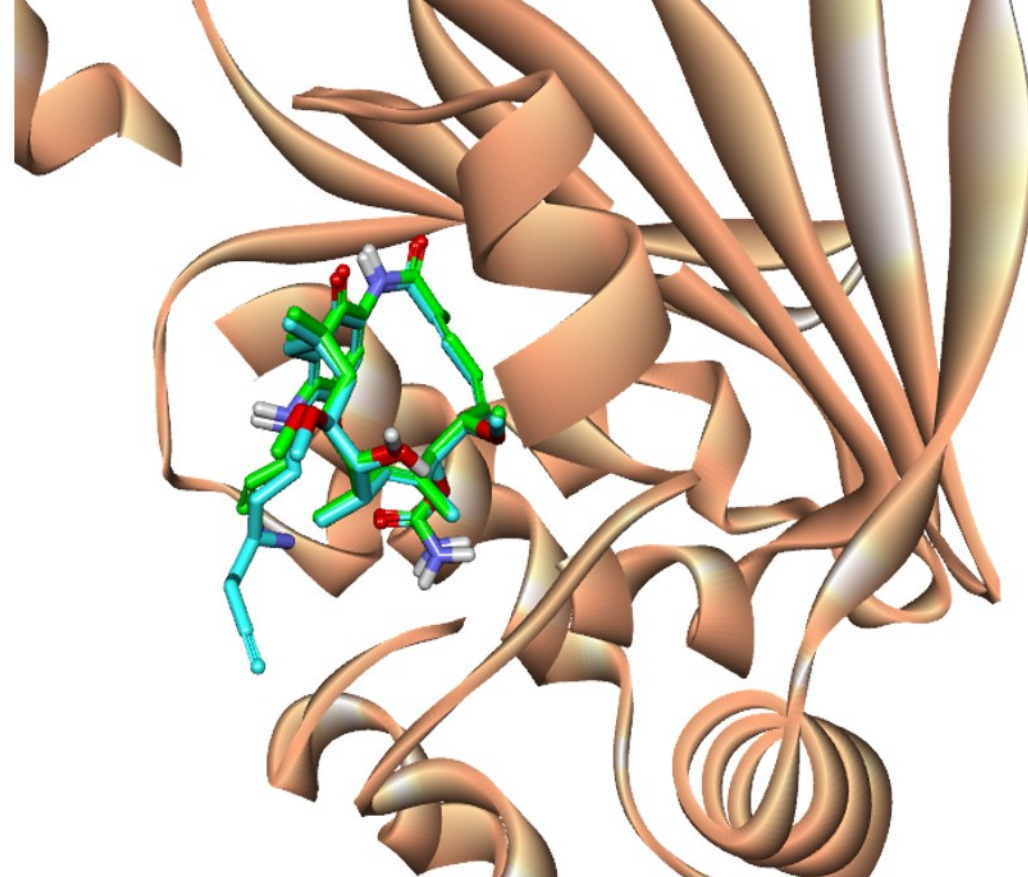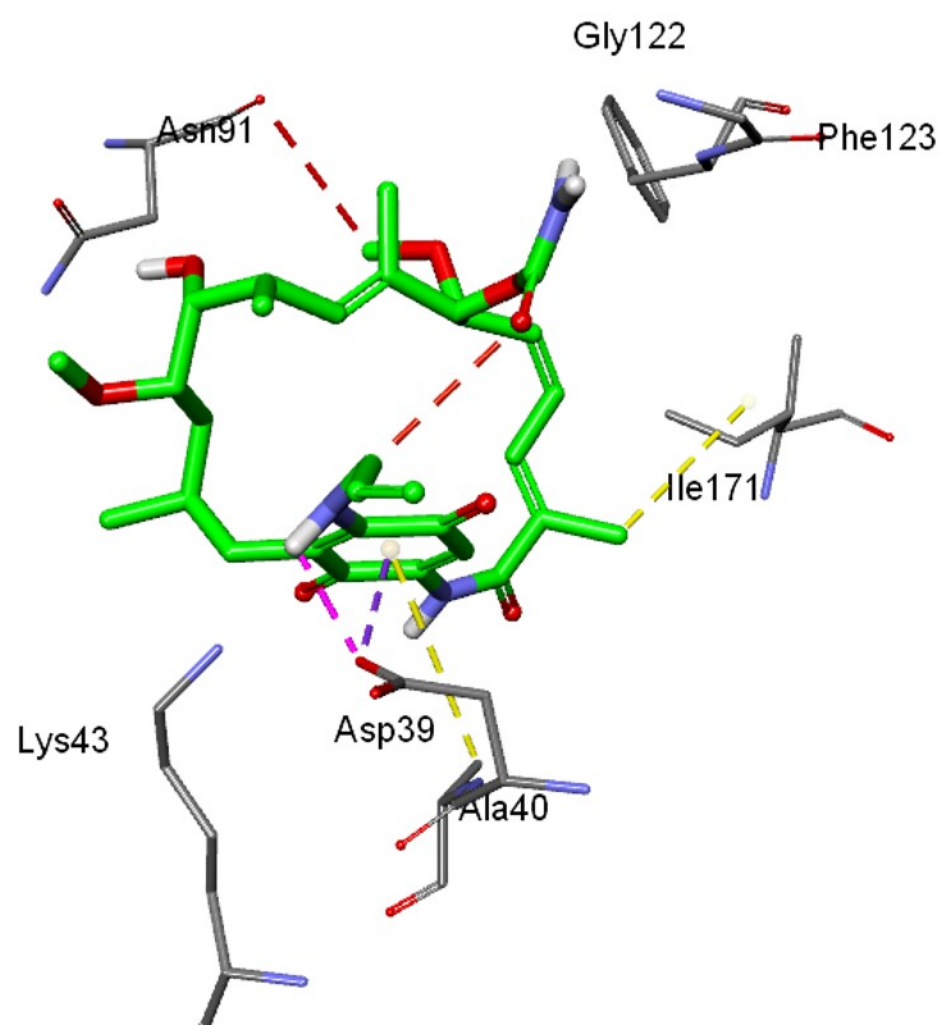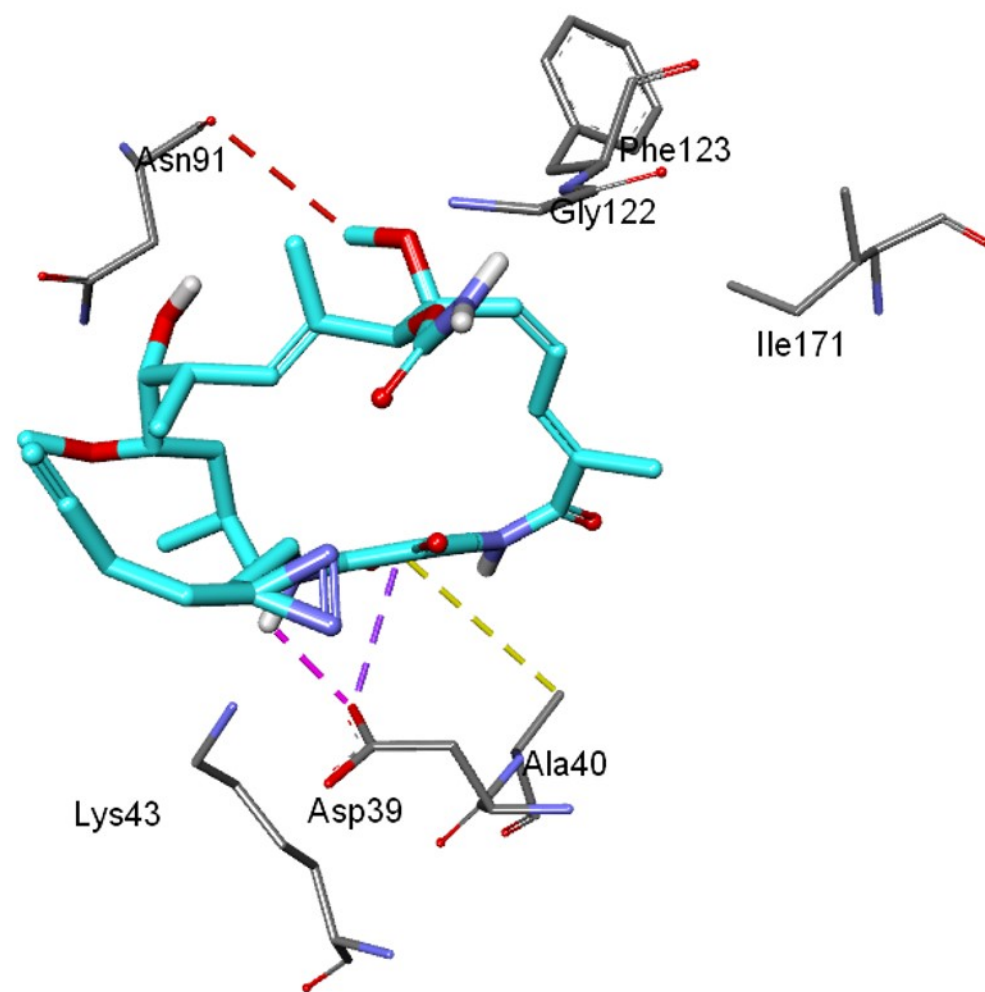

Supplement: FIG S5 [file mSystems.00089-21-sf005.pdf]
